# Tuning the Reactivity of Micellar Nanoreactors by Precise Adjustments of the Amphiphile and Substrate Hydrophobicity

**DOI:** 10.1021/acs.macromol.1c01755

**Published:** 2021-12-01

**Authors:** Shahar Tevet, Shreyas S. Wagle, Gadi Slor, Roey J. Amir

**Affiliations:** † Department of Organic Chemistry, School of Chemistry, Faculty of Exact Sciences, Tel-Aviv University, Tel-Aviv 6997801, Israel; ‡ Tel-Aviv University Center for Nanoscience and Nanotechnology, Tel-Aviv University, Tel-Aviv 6997801, Israel; § Blavatnik Center for Drug Discovery, Tel-Aviv University, Tel-Aviv 6997801, Israel; ∥ ADAMA Center for Novel Delivery Systems in Crop Protection, Tel-Aviv University, Tel-Aviv 6997801, Israel; ⊥ The Center for Physics and Chemistry of Living Systems, Tel-Aviv University, Tel-Aviv 6997801, Israel

## Abstract

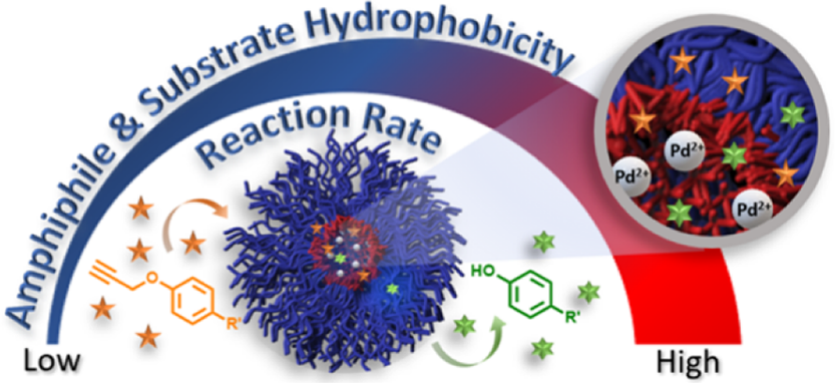

Polymeric assemblies, such as micelles, are gaining increasing attention due to their ability to serve as nanoreactors for the execution of organic reactions in aqueous media. The ability to conduct organic transformations, which have been traditionally limited to organic media, in water is essential for the further development of important fields ranging from green catalysis to bioorthogonal chemistry. Considering the recent progress that has been made to expand the range of organometallic reactions conducted using nanoreactors, we aimed to gain a deeper understanding of the roles of the hydrophobicity of both the core of micellar nanoreactors and the substrates on the reaction rates in water. Toward this goal, we designed a set of five metal-loaded micelles composed of polyethylene glycol–dendron amphiphiles and studied their ability to serve as nanoreactors for a palladium-mediated depropargylation reaction of four substrates with different log *P* values. Using dendrons as the hydrophobic block, we could precisely tune the lipophilicity of the nanoreactors, which allowed us to reveal linear correlations between the rate constants and the hydrophobicity of the amphiphiles (estimated by the dendron’s cLog *P*). While exponential dependence was obtained for the lipophilicity of the substrates, a similar degree of rate acceleration was observed due to the increase in the hydrophobicity of the amphiphiles regardless of the effect of the substrate’s log *P*. Our results demonstrate that while increasing the hydrophobicity of the substrates may be used to accelerate reaction rates, tuning the hydrophobicity of the micellar nanoreactors can serve as a vital tool for further optimization of the reactivity and selectivity of nanoreactors.

## Introduction

While an aqueous environment is essential for all living systems, using water as a solvent does not necessarily translate well for conducting organic reactions, particularly in organometallic chemistry. The limited applicability of water as a reaction medium emerges from the fact that the majority of the chemicals used in such reactions are lipophilic, in addition to the risk of poisoning of the reactive species/catalysts by water molecules. Nevertheless, the development of methodologies for conducting organic reactions in aqueous media has a significant influence on various fields, from synthetic biology and therapeutic biomaterials to green chemistry.^1−9^

Polymeric micelles can act as nanometer-sized flasks for conducting organic reactions in water.^10^ Micelles can provide the solubility and protective environment for the lipophilic reactants, shielding them from the surrounding aqueous environment.^11,12^ In recent years, significant progress in conducting organic transformation and specifically organometallic reactions in water has been reported by Lipshutz,^12−17^ Meijer,^3,18,19^ Unciti-Broceta,^5,20−24^ Zimmerman,^25−27^ O’Reilly,^28,29^ and others.^30−34^ The ability to perform organometallic reactions in aqueous media can contribute significantly to increasing the sustainability of organic synthesis by reducing the usage of organic solvents. Furthermore, the development of metal-loaded nanoreactors can open new horizons for broadening the scope of bioorthogonal approaches. Moreover, despite the great progress in this field, the rational design of micellar systems as nanoreaction vessels is still a huge chemical challenge, mostly due to the lack of broader knowledge of the structure–activity relations of these systems.

**Figure 1:**
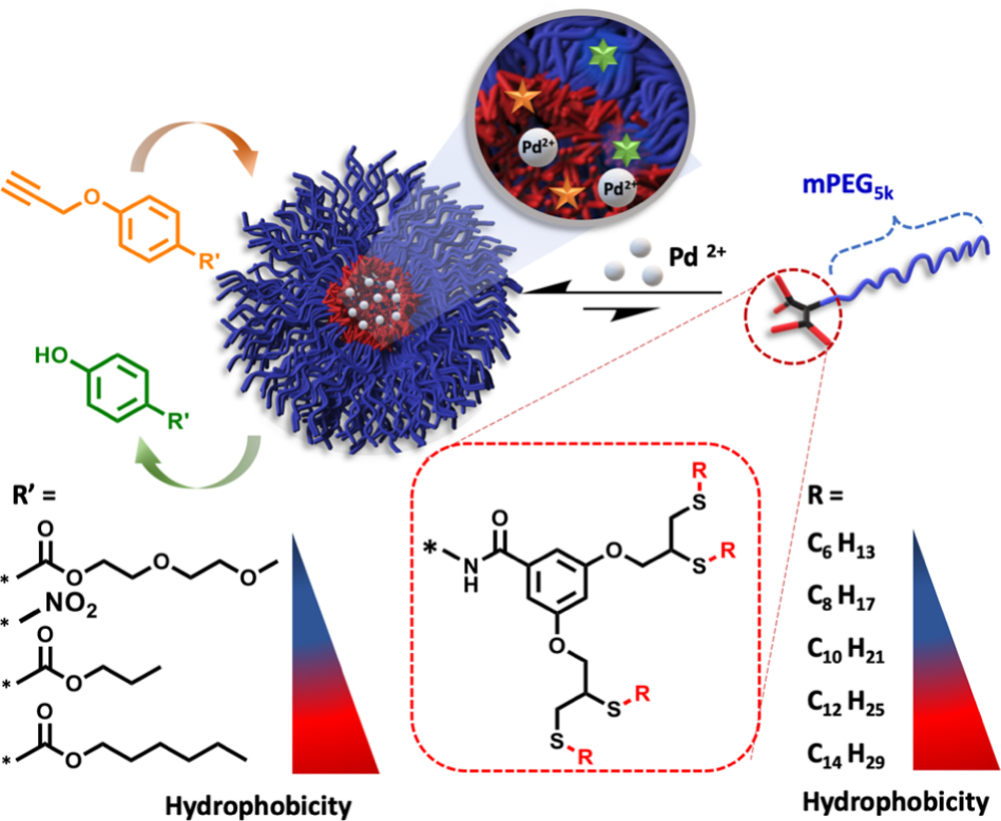
Schematic illustration of palladium-loaded micellar nanoreactors based on PEG–dendron amphiphiles for the depropargylation of substrates with increasing degrees of lipophilicity.

To gain a better understanding of the function of micellar nanoreactors as reaction media, we aimed to systematically study the influence of the hydrophobicities of both the core of the micelles and the substrate on reaction rates in water. We chose the palladium-mediated *O*-propargyl cleavage^35−44^ as a model reaction due to its potential to serve as a bioorthogonal approach for the activation of prodrugs.^4,5,7,45^ Toward this study, we developed a metal-embedded micellar system using high-molecular-precision linear-dendritic amphiphiles. Dendritic architectures have been previously utilized as nanoreactors due to their monodispersity and ability to precisely tune their structures.^46−49^ Hence, inspired by these reports and our previous studies with dendritic micelles, we envisioned that the usage of dendrons with different alkyl end-groups as the hydrophobic blocks of the amphiphiles would grant us maximal molecular control over the lipophilicity of the micellar core when studying the rates of our model reaction. To evaluate the impact of the substrate’s hydrophobicity on the reaction rate, we also synthesized low-molecular-weight propargyl-modified compounds with different degrees of lipophilicity and used them in our model depropargylation reaction. This methodology allowed us to estimate the individual impact of the lipophilic microenvironment of the micellar core and the hydrophobicity of the substrates on the reaction rate (Figure 1).

## Results and Discussion

### Molecular Design and Synthesis of Amphiphiles

**Scheme 1:**
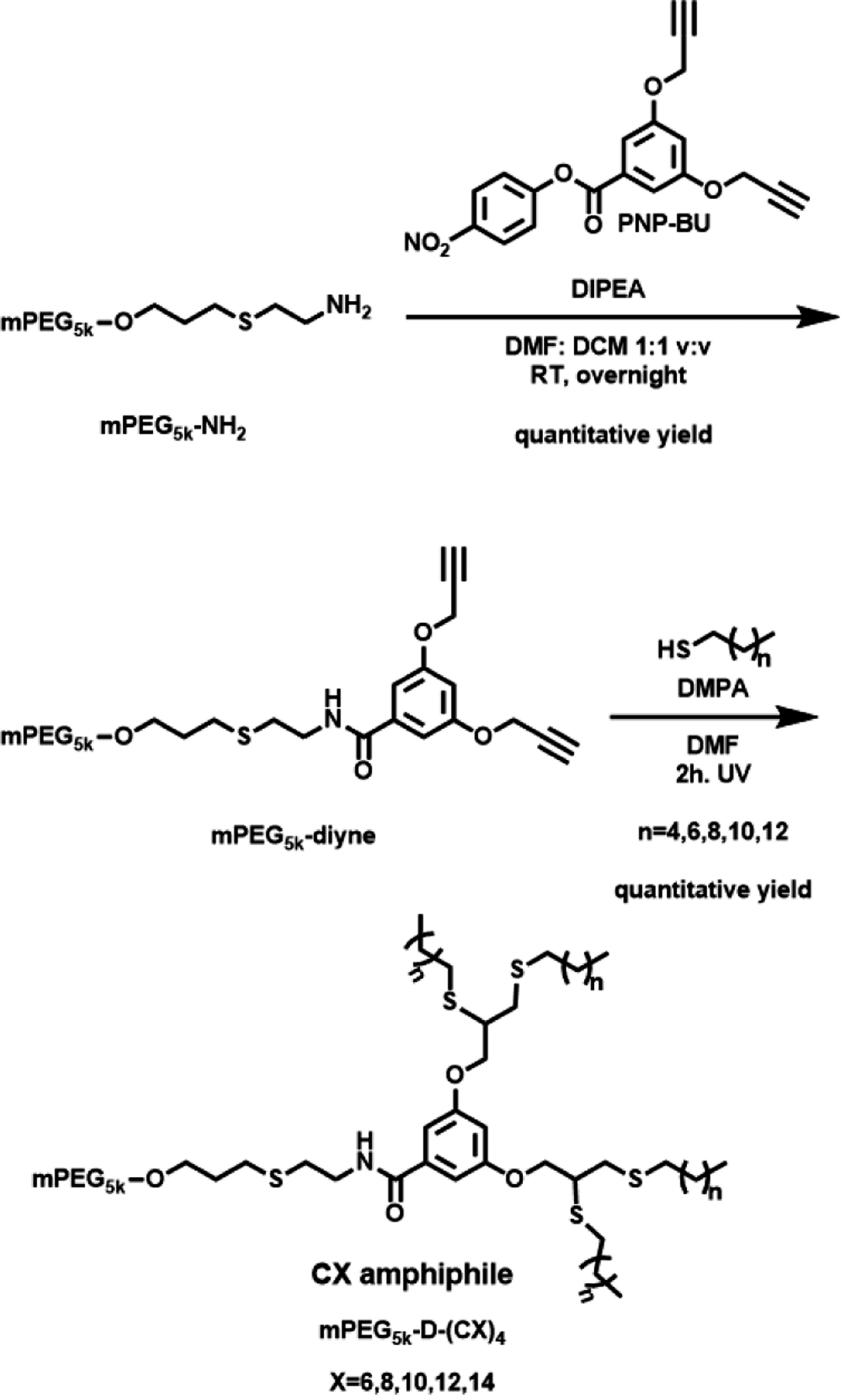
Synthetic Route for CX Amphiphilic Hybrids *X* refers to the number of carbons at the aliphatic chain.

For the micellar framework, we designed amphiphilic hybrids based on a commercial 5 kDa monofunctional polyethylene glycol (mPEG) as a hydrophilic block and a dendron with four aliphatic end-groups as the hydrophobic block. For the metal catalyst, we chose to work with a simple palladium(II) acetate salt, which has poor aqueous solubility. The hydrophobic micellar core should encapsulate the lipophilic metal salt, affording its solubilization in water and providing the required microenvironment for the organometallic reaction. Our synthetic methodology was aimed to be modular and step-efficient. In addition, it gave us high molecular control over the degree of hydrophobicity by simply tuning the length of the aliphatic end-groups. The amphiphiles were synthesized in only two high yielding steps, starting by conjugating mPEG-amine (mPEG_5k_-NH_2_) with an activated *para*-nitrophenol ester of the dipropargyl branching unit to yield a stable amide bond. The thiol-yne reaction of the dialkyne-functionalized mPEG (mPEG_5k_-diyne) with five different linear aliphatic thiols with lengths ranging from 6 to 14 carbons allowed us to produce a dendritic structure containing two 1,2 di-mercapto ether moieties in each dendron (Scheme 1). ^1^H NMR, size exclusion chromatography (SEC), high-performance liquid chromatography (HPLC), and matrix-assisted laser desorption ionization time-of-flight mass spectrometry (MALDI-TOF MS) measurements were used to verify the synthetic conversion and the product’s purity and polydispersity, and the experimental results showed excellent correlation with the expected values, as can be seen in the Supporting Information.

### Self-Assembly into Micellar Nanoreactors

Once completing the synthesis of the five amphiphiles, we wished to examine their self-assembly into micellar structures in aqueous media (phosphate buffer saline—PBS, pH 7.4, 37 °C). First, we measured their critical micelle concentration (CMC) values using the Nile red method.^50^ Based on the increase in the hydrophobicity of the dendrons due to the elongation of the aliphatic end-groups by a few carbons, we expected a slight decrease in the CMC value, as has been previously reported by us for other dendritic amphiphiles.^51,52^ As seen in Table 1 and Figures S17–S22, the CMC values for mPEG_5k_-D-(CX)_4_ amphiphiles ranged between 4 ± 1 μM for the C6 amphiphile and 3 ± 1 μM for the C14 amphiphile.

**Table 1:** Amphiphiles and Their Properties^a^

amphiphile	end-group	*M*_n_^a^(kDa)	D̵	*M*_p_^b^(kDa)	*M*_n_^c^(kDa)	CMC^d^ (μM)	*D*_H_^e^(nm)	*D*_H_^f^(nm)	cLog *P*^g^
C6	hexyl	5.0	1.08	5.8	5.8	4 ± 1	14 ± 1	12 ± 3	13.2
C8	octyl	5.6	1.04	6.0	5.9	4 ± 1	21 ± 3	13 ± 3	17.4
C10	decyl	5.9	1.05	6.1	6.0	3 ± 1	23 ± 4	15 ± 2	21.7
C12	dodecyl	6.1	1.05	6.2	6.2	3 ± 1	23 ± 5	16 ± 4	25.9
C14	tetradecyl	6.2	1.05	6.4	6.3	3 ± 1	31 ± 4	18 ± 3	30.1

a Measured by SEC using PEG commercial standards.

b Measured by MALDI-TOF MS.

c Calculated based on mPEG_5kDa_ and the expected exact mass of the dendrons.

d Determined using the Nile red method.

e Hydrodynamic diameter measured by DLS of micelles formed from amphiphiles only.

f Hydrodynamic diameter measured by DLS of micelles with the encapsulated Pd(OAc)_2_ salt.

g Calculated for only the dendritic group of the amphiphile via ChemDraw Version 18.2.

After confirming the self-assembly of nanostructures in an aqueous environment, we used dynamic light scattering (DLS) to measure the sizes of the formed structures. We determined the diameters of the assembled structures to be in the range of 14–31 nm (Table 1), which indicates that the amphiphiles self-assembled into nanosized micelles. The DLS results show that the amphiphiles with longer hydrophobic aliphatic end-groups formed larger structures, while the ones with shorter end-groups gave smaller sizes. It is interesting to note that although the change in the length of the end-groups is relatively small, a disproportional increase in the diameter was observed, indicating the increased packing and higher aggregation numbers of polymeric amphiphiles with longer aliphatic end-groups.^51^ Transmission electron microscopy (TEM) was further used to validate the formation of spherical structures with similar diameters (Figure S24).

Since the vicinal ether/thio-ether functional groups present throughout the dendritic architecture can potentially act as chelating sites for palladium,^53,54^ we wished to assess the effect of the presence of the metal salt on the nanostructures. We combined the amphiphiles and the palladium acetate salt at a 1:2 molar ratio in acetone and mixed them briefly. After the evaporation of the organic solvent, the mixture was redissolved in PBS, and DLS size measurements were performed. Interestingly, in the presence of the palladium salt, the different amphiphiles self-assembled into smaller structures with relatively similar sizes in the range of 12–18 nm (Table 1 and Figure S23). The results confirmed the formation of micelles also in the presence of the metal salt and suggested a change in the packing of the micellar structures, making them more compact, which might indicate the formation of the palladium complex within the micellar core. TEM images provided further validation of the spherical shape of the metal-loaded micelles (Figure S24).

To evaluate whether the dendritic branches of the amphiphiles can interact with the palladium ions, we performed a complexation experiment using a combination of C12 amphiphile and the palladium acetate salt. We prepared a series of solutions with different molar ratios of the metal to amphiphile, ranging between 0 and 200%, in CDCl_3_ and monitored the complex formation by ^1^H NMR (Figure S25). The use of an organic solvent was due to the limited ability to observe the signals of the protons in the hydrophobic core of the micelles in water as a result of their extremely low mobility and short relaxation times.^55,56^ The results show that upon increasing the percentage of the metal salt, the peaks of the protons near the vicinal ether/thio-ether groups were significantly broadened until entirely disappearing from the spectrum. This peak broadening is indicative of the complex formation at or near these sites, causing decreased mobility and hence short relaxations times. It is interesting to notice that besides the mentioned sites in positions α and β for the thio-ethers, the rest of the carbons of the aliphatic end-groups did not seem to be affected by the complexation.

### Studying the Effect of Hydrophobicity on the Rate of the Depropargylation Reaction

#### Tuning the Nanoreactor’s Hydrophobicity

Once the palladium-loaded micelles were characterized, we wished to examine their ability to conduct the depropargylation reaction and study the effect of increasing the hydrophobicity of the amphiphile on the reaction rate.

As a model substrate for the depropargylation reaction, we synthesized a PNP-propargyl ether (PNPPE), which upon the cleavage of the *O*-propargyl group should transform back to PNP. Importantly, the PNP product has a lower log *P* value than the PNPPE substrate (Table S1), indicative of its higher solubility in water. This may facilitate the reaction, which is likely to take place inside the hydrophobic microenvironment of the micellar core. The hydrophobic substrate will tend to migrate into the micellar structures due to its low solubility, and once transformed into the more hydrophilic product, it will be able to migrate back to the outer aqueous environment.

To prepare the metal-loaded micelles, the palladium salt and the different amphiphiles were dissolved separately in acetone, mixed, and stirred briefly, followed by the evaporation of the organic solvent and rehydration in PBS as described before. Finally, the PNPPE substrate was added, and the reaction was monitored by HPLC. A sample of the amphiphile and substrate in the absence of palladium was used as a control for monitoring the stability of the substrate solution over time and to ensure that its hydrolysis cannot be catalyzed by the micellar system alone (Figure S30). A control of the substrate and metal in the absence of the micellar structures could not be measured since both compounds have poor water solubility (Figure S31).

**Figure 2:**
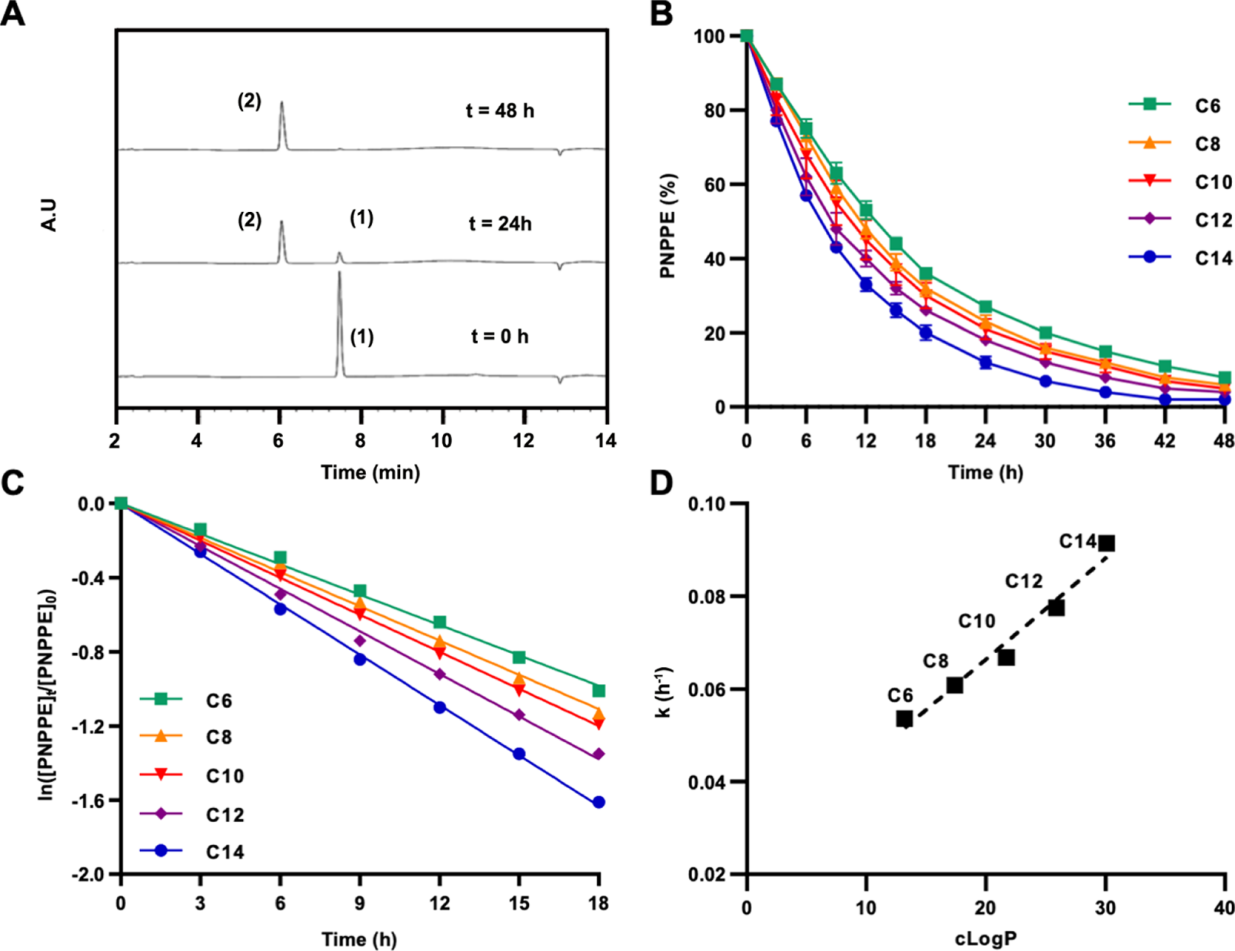
HPLC-based analysis of *O*-propargyl cleavage kinetics of the PNPPE substrate after treatment with metal-loaded micelles composed from amphiphiles with different degrees of hydrophobicity; (A) Representative HPLC chromatogram overlay (taken at 307 nm), showing the transformation of PNPPE (1) to PNP (2). [Amphiphile] = 42 μM; [Pd(OAc)_2_] = 83 μM; and [PNPPE] = 166 μM. (B) Normalized PNPPE consumption over time. (C) Natural log of the normalized experimental kinetic data. (D) cLog *P* values of the amphiphiles’ dendrons plotted against the calculated rate constants.

To evaluate the propargyl cleavage rate of the substrate, we measured the area under the curve of the substrate’s peak in each chromatogram (Figure 2A) and plotted the decrease in concentration (in %) as a function of time (Figure 2B). To evaluate the kinetics of the reaction, a natural log of the normalized experimental data was plotted against time (Figure 2C), providing a linear equation correlating with the first-order reaction ln[*A*] = −*kt* + ln[*A*]_0_. The rate constant (*k*) values were calculated based on the above first-order equation, and the theoretical half-life (*t*_1/2_) values were calculated from *t*_1/2_ = ln(*2*)/*k*. The k and *t*_1/2_ values are presented alongside the experimental value of *t*_1/2_ in Table S1. These experiments indicate that the reaction occurs faster as the aliphatic end-groups are longer, with *k* values being almost double when considering the change from the C6 to C14 amphiphile (Table S1). To ensure that the observed trend could be attributed only to the hydrophobicity of the amphiphiles, we used inductively coupled plasma (ICP)–MS to quantify the amount of palladium, which was found to be similar for all five amphiphiles, regardless of their hydrophobicity (Figure S32). These results highlight the importance of the hydrophobicity of the nanoreactor in the execution of the propargyl cleavage reaction. Furthermore, it demonstrates how small structural changes, of only a few carbons in the hydrophobic block of the amphiphiles, can have a remarkable influence on the activity of the nanoreactor.

To compare the different amphiphiles and gain an indication for the influence of the amphiphiles’ hydrophobicity on the reaction rate, the calculated *k* values were plotted against the dendrons’ cLog *P* values (Figure 2D). Although it is clear that the amphiphile’s overall hydrophobicity should be lower due to the hydrophilic PEG block, the dendrons’ cLog *P* could give us a quantitative parameter that is a key part of the total hydrophobicity of the amphiphile. The results indicate a linear correlation between the cLog *P* value of the dendrons and the reaction rates, emphasizing the importance of the hydrophobicity of the nanoreactors in the execution of the reaction.

**Figure 3:**
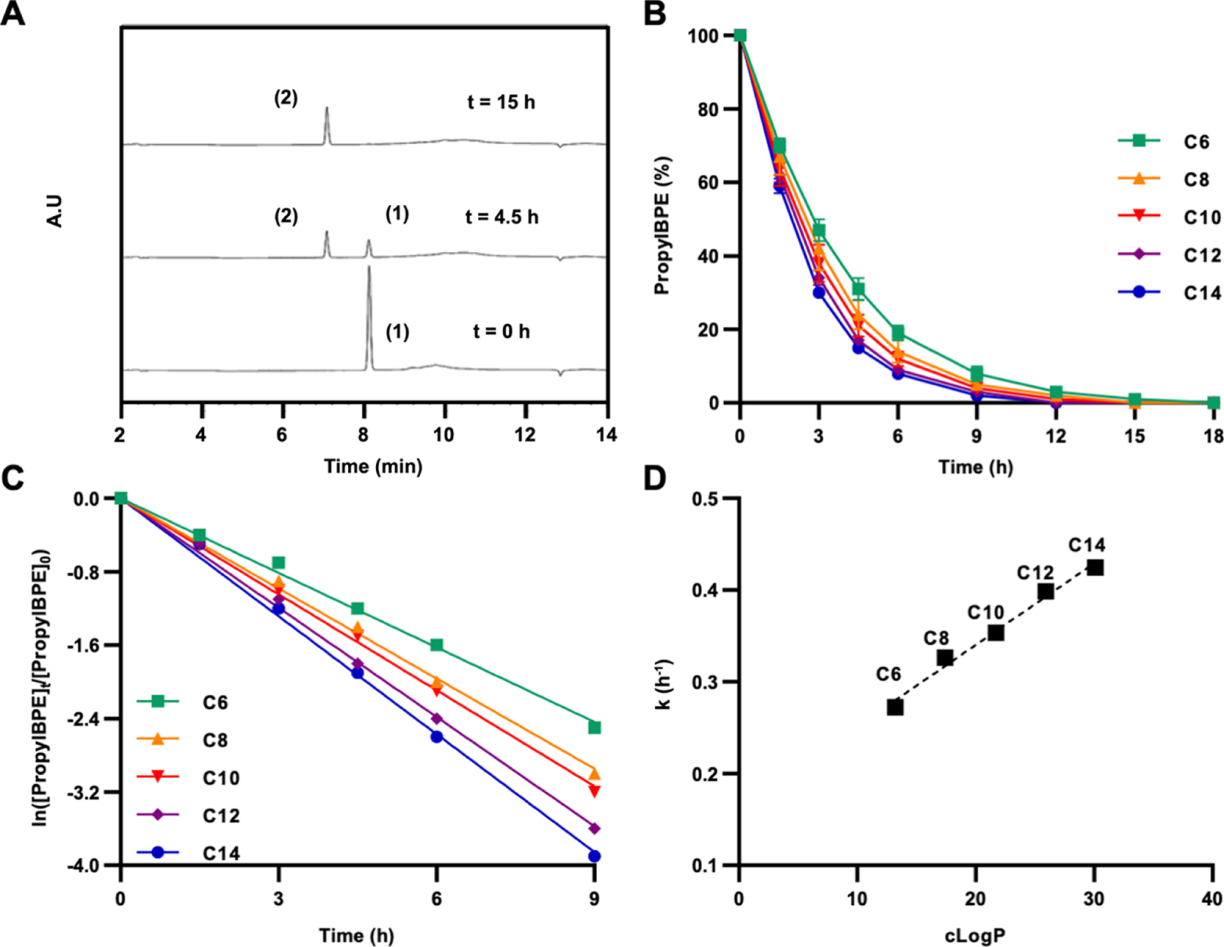
*O*-propargyl cleavage profile of the PropylBPE substrate after treatment with metallic micelles composed from amphiphiles with different degrees of hydrophobicity; (A) Representative HPLC chromatogram overlay (taken at 252 nm), showing the transformation of the propyl substrate (1) to its depropargylated product (2). [Amphiphile] = 42 μM; [Pd(OAc)2] = 83 μM; and [PropylBPE] = 100 μM. (B) Normalized propyl consumption over time. (C) Natural log of the normalized experimental kinetic data. (D) cLog *P* values of the amphiphiles’ dendrons plotted against their corelated calculated rate constant.

After observing the linear influence of the hydrophobicity of the nanoreactor on the propargyl cleavage reaction for the PNPPE substrate, we wished to repeat these experiments using a different propargyl-containing substrate. We decided to synthesize 4-(propargyloxy)benzoic acid propyl ester (PropylBPE) in order to examine whether the kinetic trends would be preserved or were specific to the PNPPE substrate. The PropylBPE substrate was incubated with all five different metal-containing micelles, following the same protocol as that described above, and the reaction was monitored by HPLC. The kinetic results (Figure 3) demonstrated a remarkably similar trend to that obtained for the propargyl cleavage for PNPPE, showing a linear correlation between the calculated k values and the cLog *P* values of the dendrons. Thus, indicating that the effect of the nanoreactor’s hydrophobicity on the reaction rate is not limited to a specific substrate. Although the linear trend was preserved, we have noticed that the reaction rates were faster for the PropylBPE substrate (Table S1). The propyl-based substrate is more lipophilic and has a higher Log *P* value than the PNPPE substrate, which indicates its higher tendency to migrate into the hydrophobic core of the micelles, resulting in a higher reaction rate. The higher *k* values for the PropylBPE substrate than that for the PNPPE substrate imply that the hydrophobicity of the substrate has an additional effect on the reaction rate in the presence of the micellar nanoreactors.

#### Adjusting the Substrate Hydrophobicity

**Figure 4:**
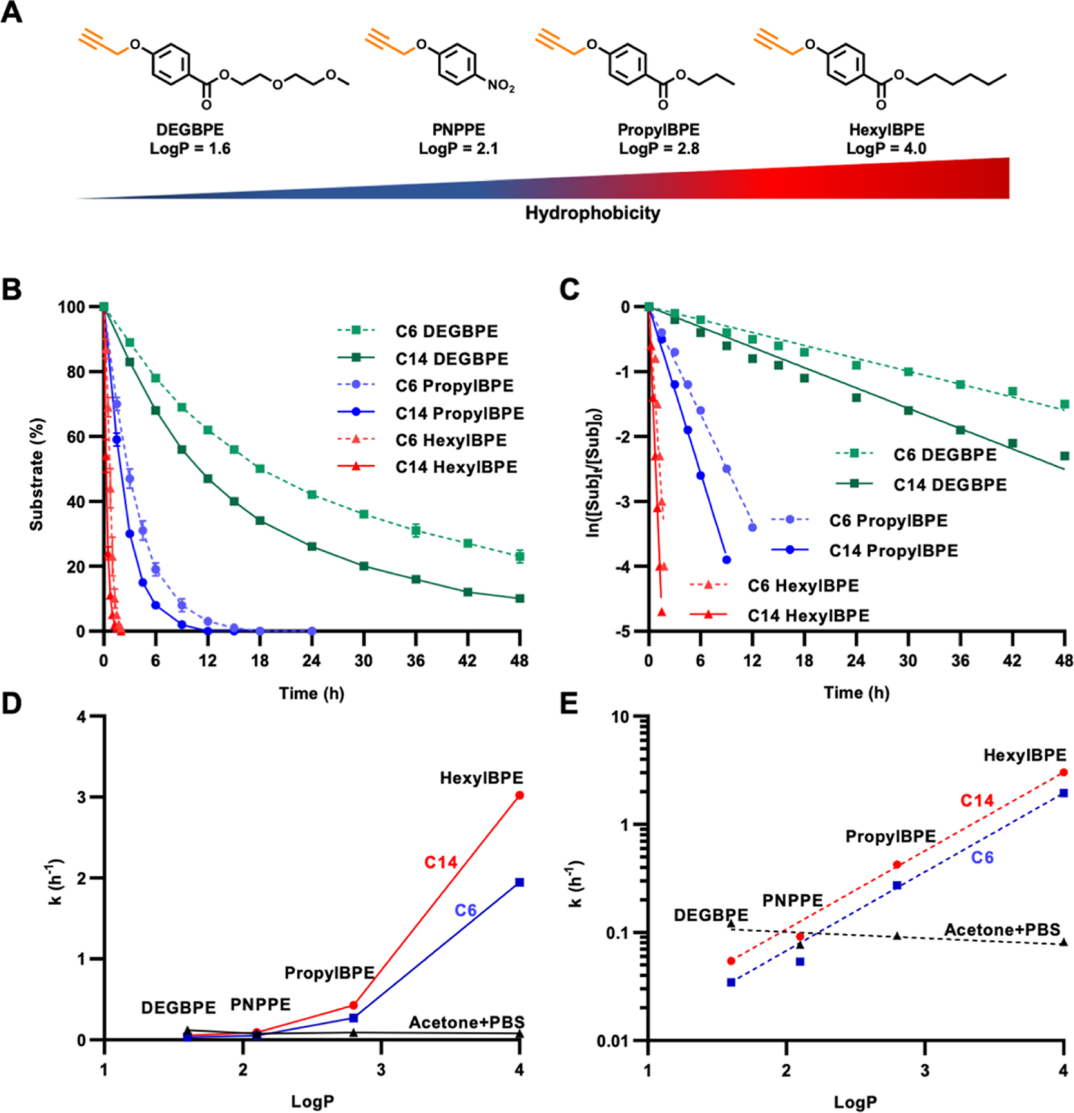
(A) Structures of substrates for the depropargylation reaction and *O*-propargyl cleavage profiles of HexylBPE, PropylBPE, PNPPE, and DEGBPE substrates; (B) Normalized substrate consumption over time in the presence of C6 (dashed lines) and C14 (full lines) metallic micelles. [Amphiphile] = 42 μM; [Pd(OAc)_2_] = 83 μM; and [Substrate] = 100 μM. (C) Natural log of the normalized experimental kinetic data. (D) Calculated rate constants values in the presence of C6 micelles (red), C14 micelles (blue), and the acetone/PBS (1:1 v/v) setup (black) plotted against the substrate Log *P* values. (E) Logarithmic representation of graph D.

Previous reports by Neumann,^57^ Escuder,^58^ and others have shown rate acceleration upon increasing the hydrophobicity of reaction substrates using catalytic hydrogel-based systems. To evaluate how the hydrophobicity of the substrate will affect the reaction rate in our micellar nanoreactors, we decided to design two additional derivatives of the propargyl-containing substrate, which would have either higher or lower lipophilicity. The two additional substrates were synthesized by replacing the propyl-ester group with either hexyl or diethylene glycol (DEG) ester, yielding HexylBPE and DEGBPE substrates, respectively (Figure 4A).

To examine the effect of the hydrophobicity of the substrate, HexylBPE and DEGBPE were incubated with C6- and C14-based micellar nanoreactors, following the same protocol as that described above. The propargyl cleavage profiles and reaction rates of the different substrates are presented in Figure 4B,C and Table S1, along with the kinetic data acquired for the propyl-based substrate.

The results show a clear trend, where the more hydrophobic substrates reacted faster, indicating the significant impact of this factor on the reaction rate. The faster reaction rate can be contributed to the higher tendency of the substrates to migrate into the micellar nanoreactor as their lipophilicity increases, as previously reported for other polymeric nanoreactors^25,26^ as well as for other catalytic systems such as hydrogels.^57^

To estimate the relative effect of the substrate lipophilicity on the reaction rates, the calculated *k* values for all four substrates in the presence of both C6 and C14 catalytic micelles were plotted against the substrates’ Log *P* values (Figure 4D). When comparing the contributions of the substrate and micellar nanoreactor hydrophobicity, it appears that while the amphiphile cLog *P* values showed a linear correlation with the reaction rates (Figures 2D and 3D), the substrate’s Log *P* values yielded an exponential correlation (Figure 4D,E). These results, which show nearly a 2-fold acceleration of the reaction rates upon switching from C6- to C14-based amphiphiles and around a 50-fold increase in the reaction rate considering the change from the least hydrophobic DEGBPE to the most hydrophobic HexylBPE, suggest that the substrate lipophilicity plays a more significant role in determining the rate of the reaction. Importantly, when comparing the kinetic data for the different substrates with the C6- and C14-based nanoreactors, similar slopes were observed when plotting the k values in a logarithmic scale against the substrates’ Log *P* value (Figure 4E). These results indicate that the amphiphilicity of the nanoreactors maintained its relative impact on the reactivity even when the lipophilicity of the substrates drastically changed. Curious to see the role of the palladium source on the reaction rates, we used a soluble Na_2_PdCl_4_ salt and tested its reactivity for all four substrates in the presence of the C14 amphiphile (Figure S33). The results showed similar kinetic rates to that of the water-insoluble Pd(OAc)_2_, providing further indication of the ability of the micellar nanoreactors to complex the palladium ions as well as the occurrence of the depropargylation reaction inside the micellar core.

To ensure that the observed kinetic trends are not the result of specific metal–substrate interactions, which are independent of the hydrophobic microenvironment of the micellar nanoreactors, we have performed a control experiment in the absence of the micelles. Instead of the micellar setup, we used a mixture of acetone/PBS (1:1 v/v) as the reaction medium, whereas the concentrations of the palladium acetate salt and the different substrates were kept the same as those for the micellar experiments. All four substrates showed relatively similar reaction rates in the range 0.08–0.12 h^–1^ (Table S1), which are of the same order of magnitude as that of the PNPPE substrate in the presence of the micellar nanoreactors (Figures 4D,E and S34). The similar rates observed for all substrates in the control experiment together with the slower reaction rates for the more hydrophilic substrate DEGBPE in the presence of the micelles provide additional support for the occurrence of the catalytic activity inside the micellar nanoreactors. Importantly, these results, similar to those of the previously reported catalytic nanoparticles, demonstrate yet again the high favorability of the micellar nanoreactors toward more hydrophobic substrates. Nevertheless, the observed kinetic trends emphasize the potential of tuning the reactivity by solely adjusting the hydrophobicity of the nanoreactors even in cases where the intrinsic hydrophobicity of the given substrate is dictated by its specific structure.

## Conclusions

In this work, we wished to study how the hydrophobicity of micellar nanoreactors would affect the rate of an organometallic reaction being executed in water. As a model reaction, we selected the bioorthogonal palladium-mediated depropargylation reaction, which has potential for biomedical and therapeutic applications such as the activation of probe molecules and prodrugs. Our nanoreactor platform was based on palladium-loaded micelles composed of PEG-dendron amphiphiles. To systematically evaluate the influence of changes in the lipophilicity of the nanoreactor on the reaction rates, we synthesized a small library of five amphiphiles and precisely tuned their hydrophobicity by varying the length of their aliphatic end-groups. We then tested the reactivity of our PEG–dendron-based nanoreactors using four propargyl-containing substrates with increasing degrees of hydrophobicity.

Kinetic analysis of the experimental data highlighted the crucial effect that the hydrophobicity of the nanoreactor have on the reaction rate as the more lipophilic amphiphiles yielded faster kinetics. The high molecular precision of our dendritic amphiphiles allowed us to demonstrate that the addition of only a few carbons to the hydrophobic block can make a significant impact on enhancing the reactivity of the nanoreactors. Interestingly, a linear correlation between the cLog *P* values of the dendritic block and the reaction rate was found. This linear correlation was observed for two different substrates, indicating that it is not limited to a specific type of substrate. In addition, the hydrophobicity of the substrate as estimated by their Log *P* showed an exponential correlation with the observed reaction rates, similar to those of the previously reported polymer-based catalytic systems. Importantly, the relative impact of the nanoreactors lipophilicity on the reaction rates remained nearly constant for all four substrates regardless of the exponential rate acceleration attributed to the hydrophobicity of the substrates.

The obtained results together with the kinetics for a control reaction medium composed of an acetone/water mixture, which showed similar rates for all substrates, highlight the high selectivity of the micellar nanoreactors toward the more hydrophobic substrates. Understanding the mutual contributions of the hydrophobicity of both the substrate and the micellar microenvironment on the reaction rate provides essential knowledge toward the rational design of nanoreactor systems, for fields ranging from green chemistry to therapeutic applications involving bioorthogonal catalysis for the activation of prodrugs in living systems.

## Supporting Information Available

Synthetic procedures, amphiphile and micelle characterization data, and detailed experimental protocols and control experiments (PDF)

## Abbreviations

CMC: critical micelle concentration
DLS: dynamic light scattering
HPLC: high-pressure liquid chromatography

## References

[ref1] Wang J.; Cheng B.; Li J.; Zhang Z.; Hong W.; Chen X.; Chen P. R. Angew. Chem., Int. Ed. 2015, 54, 5364–5368. 10.1002/anie.201409145. 25765364

[ref2] Szponarski M.; Schwizer F.; Ward T. R.; Gademann K. Chem. Commun. 2018, 1, 84 10.1038/s42004-018-0087-y.

[ref3] Liu Y.; Pujals S.; Stals P. J. M.; Paulöhrl T.; Presolski S. I.; Meijer E. W.; Albertazzi L.; Palmans A. R. A. J. Am. Chem. Soc. 2018, 140, 3423–3433. 10.1021/jacs.8b00122. 29457449 PMC5997400

[ref4] Weiss J. T.; Dawson J. C.; Macleod K. G.; Rybski W.; Fraser C.; Torres-Sánchez C.; Patton E. E.; Bradley M.; Carragher N. O.; Unciti-Broceta A. Nat. Commun. 2014, 5, 3277 10.1038/ncomms4277. 24522696 PMC3929780

[ref5] Yusop R. M.; Unciti-Broceta A.; Johansson E. M. V.; Sánchez-Martín R. M.; Bradley M. Nat. Chem. 2011, 3, 239–243. 10.1038/nchem.981. 21336331

[ref6] Tonga G. Y.; Jeong Y.; Duncan B.; Mizuhara T.; Mout R.; Das R.; Kim S. T.; Yeh Y.-C.; Yan B.; Hou S.; Rotello V. M. Nat. Chem. 2015, 7, 597–603. 10.1038/nchem.2284. 26100809 PMC5697749

[ref7] Unciti-Broceta A.; Johansson E. M. V.; Yusop R. M.; Sánchez-Martín R. M.; Bradley M. Nat. Protoc. 2012, 7, 1207–1218. 10.1038/nprot.2012.052. 22653159

[ref8] Gallou F.; Isley N. A.; Ganic A.; Onken U.; Parmentier M. Green Chem. 2016, 18, 14–19. 10.1039/c5gc02371h.

[ref9] Tu J.; Xu M.; Franzini R. M. ChemBioChem 2019, 20, 1615–1627. 10.1002/cbic.201800810. 30695126

[ref10] C&EN Global Enterprise, 2020; Vol. 98, pp 20–23.

[ref11] Lipshutz B. H. Johnson Matthey Technol. Rev. 2017, 61, 196–202. 10.1595/205651317x695785.

[ref12] Lipshutz B. H.; Ghorai S.; Abela A. R.; Moser R.; Nishikata T.; Duplais C.; Krasovskiy A.; Gaston R. D.; Gadwood R. C. J. Org. Chem. 2011, 76, 4379–4391. 10.1021/jo101974u. 21548658 PMC3608414

[ref13] Pang H.; Hu Y.; Yu J.; Gallou F.; Lipshutz B. H. J. Am. Chem. Soc. 2021, 143, 3373–3382. 10.1021/jacs.0c11484. 33630579

[ref14] Cortes-Clerget M.; Akporji N.; Zhou J.; Gao F.; Guo P.; Parmentier M.; Gallou F.; Berthon J. Y.; Lipshutz B. H. Nat. Commun. 2019, 10, 2169 10.1038/s41467-019-09751-4. 31092815 PMC6520378

[ref15] Takale B. S.; Thakore R. R.; Casotti G.; Li X.; Gallou F.; Lipshutz B. H. Angew. Chem., Int. Ed. 2021, 60, 4158–4163. 10.1002/anie.202014141. 33180988

[ref16] Lipshutz B. H.; Aguinaldo G. T.; Ghorai S.; Voigtritter K. Org. Lett. 2008, 10, 1325–1328. 10.1021/ol800028x. 18335947

[ref17] Bu M.-j.; Cai C.; Gallou F.; Lipshutz B. H. Green Chem. 2018, 20, 1233–1237. 10.1039/c7gc03866f.

[ref18] Artar M.; Souren E. R. J.; Terashima T.; Meijer E. W.; Palmans A. R. A. ACS Macro Lett. 2015, 4, 1099–1103. 10.1021/acsmacrolett.5b00652. 35614811

[ref19] Liu Y.; Turunen P.; De Waal B. F. M.; Blank K. G.; Rowan A. E.; Palmans A. R. A.; Meijer E. W. Mol. Syst. Des. Eng. 2018, 3, 609–618. 10.1039/c8me00017d.

[ref20] Weiss J. T.; Carragher N. O.; Unciti-Broceta A. Sci. Rep. 2015, 5, 9329 10.1038/srep09329. 25788464 PMC4365405

[ref21] Pérez-López A. M.; Rubio-Ruiz B.; Valero T.; Contreras-Montoya R.; Álvarez de Cienfuegos L.; Sebastián V.; Santamaría J.; Unciti-Broceta A. J. Med. Chem. 2020, 63, 9650–9659. 32787091 10.1021/acs.jmedchem.0c00781 PMC7497487

[ref22] Sancho-Albero M.; Rubio-Ruiz B.; Pérez-López A. M.; Sebastián V.; Martín-Duque P.; Arruebo M.; Santamaría J.; Unciti-Broceta A. Nat. Catal. 2019, 2, 864–872. 10.1038/s41929-019-0333-4. 31620674 PMC6795537

[ref23] van de L’Isle M. O. N.; Ortega-Liebana M. C.; Unciti-Broceta A. Curr. Opin. Chem. Biol. 2021, 61, 32–42. 33147552 10.1016/j.cbpa.2020.10.001

[ref24] Rubio-Ruiz B.; Weiss J. T.; Unciti-Broceta A. J. Med. Chem. 2016, 59, 9974–9980. 10.1021/acs.jmedchem.6b01426. 27786474

[ref25] Chen J.; Wang J.; Bai Y.; Li K.; Garcia E. S.; Ferguson A. L.; Zimmerman S. C. J. Am. Chem. Soc. 2018, 140, 13695–13702. 10.1021/jacs.8b06875. 30192530

[ref26] Chen J.; Wang J.; Li K.; Wang Y.; Gruebele M.; Ferguson A. L.; Zimmerman S. C. J. Am. Chem. Soc. 2019, 141, 9693–9700. 10.1021/jacs.9b04181. 31124359 PMC13229381

[ref27] Bai Y.; Feng X.; Xing H.; Xu Y.; Kim B. K.; Baig N.; Zhou T.; Gewirth A. A.; Lu Y.; Oldfield E.; Zimmerman S. C. J. Am. Chem. Soc. 2016, 138, 11077–11080. 10.1021/jacs.6b04477. 27529791

[ref28] Cotanda P.; Lu A.; Patterson J. P.; Petzetakis N.; O’Reilly R. K. Macromolecules 2012, 45, 2377–2384. 10.1021/ma2027462.

[ref29] Lestini E.; Blackman L. D.; Zammit C. M.; Chen T.; Williams R. J.; Inam M.; Couturaud B.; O’Reilly R. K. Polym. Chem. 2018, 9, 820–823. 10.1039/c7py02050c.

[ref30] Ansari T. N.; Taussat A.; Clark A. H.; Nachtegaal M.; Plummer S.; Gallou F.; Handa S. ACS Catal. 2019, 9, 10389–10397. 10.1021/acscatal.9b02622.

[ref31] Bihani M.; Ansari T. N.; Finck L.; Bora P. P.; Jasinski J. B.; Pavuluri B.; Leahy D. K.; Handa S. ACS Catal. 2020, 10, 6816–6821. 10.1021/acscatal.0c01196.

[ref32] Sabatino V.; Rebelein J. G.; Ward T. R. J. Am. Chem. Soc. 2019, 141, 17048–17052. 10.1021/jacs.9b07193. 31503474 PMC6823642

[ref33] Villarino L.; Chordia S.; Alonso-Cotchico L.; Reddem E.; Zhou Z.; Thunnissen A. M. W. H.; Maréchal J.-D.; Roelfes G. ACS Catal. 2020, 10, 11783–11790. 10.1021/acscatal.0c01619. 33101759 PMC7574625

[ref34] Destito P.; Sousa-Castillo A.; Couceiro J. R.; López F.; Correa-Duarte M. A.; Mascareñas J. L. Chem. Sci. 2019, 10, 2598–2603. 10.1039/c8sc04390f. 30996975 PMC6419927

[ref35] Pal M.; Parasuraman K.; Yeleswarapu K. R. Org. Lett. 2003, 5, 349–352. 10.1021/ol027382t. 12556189

[ref36] Patterson D. M.; Nazarova L. A.; Prescher J. A. ACS Chem. Biol. 2014, 9, 592–605. 10.1021/cb400828a. 24437719

[ref37] Ramil C. P.; Lin Q. Chem. Commun. 2013, 49, 11007–11022. 10.1039/c3cc44272a. PMC3847904 24145483

[ref38] Sletten E. M.; Bertozzi C. R. Angew. Chem., Int. Ed. 2009, 48, 6974–6998. 10.1002/anie.200900942. PMC2864149 19714693

[ref39] Bertozzi C. R. Acc. Chem. Res. 2011, 44, 651–653. 10.1021/ar200193f. 21928847 PMC4408923

[ref40] Devaraj N. K. ACS Cent. Sci. 2018, 4, 952–959. 10.1021/acscentsci.8b00251. 30159392 PMC6107859

[ref41] Hang H. C.; Yu C.; Kato D. L.; Bertozzi C. R. Proc. Natl. Acad. Sci. U.S.A. 2003, 100, 14846–14851. 10.1073/pnas.2335201100. 14657396 PMC299823

[ref42] Li J.; Chen P. R. Nat. Chem. Biol. 2016, 12, 129–137. 10.1038/nchembio.2024. 26881764

[ref43] Rambabu D.; Bhavani S.; Swamy N. K.; Basaveswara Rao M. V.; Pal M. Tetrahedron Lett. 2013, 54, 1169–1173. 10.1016/j.tetlet.2012.12.093.

[ref44] Coelho S. E.; Schneider F. S. S.; De Oliveira D. C.; Tripodi G. L.; Eberlin M. N.; Caramori G. F.; De Souza B.; Domingos J. B. ACS Catal. 2019, 9, 3792–3799. 10.1021/acscatal.9b00210.

[ref45] Huang R.; Li C.-H.; Cao-Milán R.; He L. D.; Makabenta J. M.; Zhang X.; Yu E.; Rotello V. M. J. Am. Chem. Soc. 2020, 142, 10723–10729. 10.1021/jacs.0c01758. 32464057 PMC7339739

[ref46] Helms B.; Liang C. O.; Hawker C. J.; Fréchet J. M. J. Macromolecules 2005, 38, 5411–5415. 10.1021/ma050701m.

[ref47] Diallo A. K.; Boisselier E.; Liang L.; Ruiz J.; Astruc D. Chem.—Eur. J. 2010, 16, 11832–11835. 10.1002/chem.201002014. 20821770

[ref48] Shema-Mizrachi M.; Pavan G. M.; Levin E.; Danani a.; Lemcoff N. G. J. Am. Chem. Soc. 2011, 133, 14359–14367. 10.1021/ja203690k. 21812463

[ref49] Gitsov I. J. Polym. Sci., Part A: Polym. Chem. 2008, 46, 5295–5314. 10.1002/pola.22828.

[ref50] Gillies E. R.; Jonsson T. B.; Fréchet J. M. J. J. Am. Chem. Soc. 2004, 126, 11936–11943. 10.1021/ja0463738. 15382929

[ref51] Segal M.; Avinery R.; Buzhor M.; Shaharabani R.; Harnoy A. J.; Tirosh E.; Beck R.; Amir R. J. J. Am. Chem. Soc. 2017, 139, 803–810. 10.1021/jacs.6b10624. 27990807

[ref52] Harnoy A. J.; Buzhor M.; Tirosh E.; Shaharabani R.; Beck R.; Amir R. J. Biomacromolecules 2017, 18, 1218–1228. 10.1021/acs.biomac.6b01906. 28267318

[ref53] Stenton B. J.; Oliveira B. L.; Matos M. J.; Sinatra L.; Bernardes G. J. L. Chem. Sci. 2018, 9, 4185–4189. 10.1039/c8sc00256h. 29780549 PMC5941270

[ref54] Yan Q.; Zheng L.; Li M.; Chen Y. J. Catal. 2019, 376, 101–105. 10.1016/j.jcat.2019.07.004.

[ref55] Buzhor M.; Avram L.; Frish L.; Cohen Y.; Amir R. J. J. Mater. Chem. B 2016, 4, 3037–3042. 10.1039/c5tb02445e. 32263042

[ref56] Wang H.; Raghupathi K. R.; Zhuang J.; Thayumanavan S. ACS Macro Lett. 2015, 4, 422–425. 10.1021/acsmacrolett.5b00199. 25949857 PMC4416465

[ref57] Haimov A.; Neumann R. J. Am. Chem. Soc. 2006, 128, 15697–15700. 10.1021/ja064294l. 17147379

[ref58] Berdugo C.; Miravet J. F.; Escuder B. Chem. Commun. 2013, 49, 10608–10610. 10.1039/c3cc45623d. 24098889

