# Supplementary material for: Tuning the Reactivity of Micellar Nanoreactors by Precise Adjustments of the Amphiphile and Substrate Hydrophobicity
Source: Macromolecules. 2021 Dec 1;54(24):11419–26. doi: 10.1021/acs.macromol.1c01755 (PMC8717824; doi:10.1021/acs.macromol.1c01755)
Supplement: Supplementary file 1 — ma1c01755_si_001.pdf [file ma1c01755_si_001.pdf]

Supplementary Information

Tuning The Reactivity Of Micellar Nanoreactors By Precise Adjustments Of The Amphiphiles And Substrates Hydrophobicity

Shahar Tevet‡[1,2], Shreyas Shankar Wagle‡[1,2], Gadi Slor[1,2] and Roey J. Amir[1,2,3,4,5]*

1 Department of Organic Chemistry, School of Chemistry, Faculty of Exact Sciences, Tel-Aviv University, Tel-Aviv 6997801, Israel

2 Tel-Aviv University Center for Nanoscience and Nanotechnology, Tel-Aviv University, Tel-Aviv 6997801, Israel

3 BLAVATNIK Center for Drug Discovery, Tel-Aviv University, Tel-Aviv 6997801, Israel

4 ADAMA Center for Novel Delivery Systems in Crop Protection, Tel-Aviv University, Tel-Aviv 6997801, Israel

5 The Center for Physics and Chemistry of Living Systems, Tel-Aviv University, Tel-Aviv 6997801, Israel

Table of Contents

Instrumentation and Materials **.......... 3**

Instrumentation 3

Materials 3

Synthesis **.......... 4**

Synthesis of mPEG$_{5k}$-D-(CX)$_4$ amphiphilic hybrids 4

Synthesis of Propargylated substrates 10

Characterization of PEG-dendron hybrids **.......... 15**

HPLC measurements 15

Size exclusion chromatography (SEC) 17

Characterization of PEG-dendron micellar structures **.......... 18**

Critical micelles' concentration (CMC) 18

Dynamic light scattering 21

TEM imaging 22

Evaluation of complex formation **.......... 23**

Depropargylation experiments **.......... 25**

General protocol for the preparation of palladium-embedded micellar nanoreactors and depropargylation experiments setup 25

ICP-MS 30

General protocol for the Na_2PdCl_4 palladium mediated depropargylation reaction experiments setup 31

General protocol for the palladium mediated depropargylation reaction in Acetone:PBS mixture experiments setup 31

References **.......... 33**

Instrumentation and Materials:

Instrumentation:

HPLC: All measurements were recorded on a Waters Alliance e2695 separations module equipped with a Waters 2998 photodiode array detector. All solvents were purchased from Bio-Lab Chemicals and were used as received. All solvents are HPLC grade. **^{1}H and ^{13}C NMR:** Spectra were recorded on Bruker Avance I and Avance III 400 MHz/100 MHz spectrometers as indicated. Chemical shifts are reported in ppm and referenced to the solvent. **SEC:** All measurements were recorded on Viscotek GPCmax by Malvern using refractive index detector and PEG standards (purchased from Sigma-Aldrich) were used for calibration. **Fluorescence spectra:** All measurements were recorded on a TECAN Infinite M200Pro device. **MALDI-TOF MS:** Analysis was conducted on a Bruker AutoFlex MALDI-TOF MS (Germany). α-Cyano-4-hydroxycinnamic acid matrix was used. **LCMS**: Measurements were conducted on a LCMS Xevo–TQD and analysis on Agilent 1260 system with single quadrupole MSD featured with multimode (ESI+APCI) ionization chamber. **TEM**: Images were taken by a JEM-1400PlusTEM at 80 kV. **ICP-MS:** Measurements were conducted on Agilent 7800 ICP-MS equipped with SPS4 Autosampler. **DLS**: All measurements were recorded on a Corduan Technology VASCOγ particle size analyzer.

Materials:

Poly (Ethylene Glycol) methyl ether (M_n=5kDa), 2,2-dimethoxy-2-phenylacetophenone (DMPA, 99%), allyl bromide, propargyl bromide (80% in toluene), 4-dimethylaminopyridine (4-DMAP, 99%), 4-Nitrophenol (99.5%), 1-(3-Dimethylaminopropyl)-3-ethylcarbodiimide hydrochloride (EDC), N,N'-dicyclohexylcarbodiimide (DCC, 99%), 2-(2-Methoxyethoxy)ethanol, 1-alkanethiols (6, 8, 12 and 14 carbons), propyl alcohol, palladium acetate and Sephadex® LH20 were purchased from Sigma-Aldrich. Cysteamine hydrochloride (98%), potassium hydroxide and Diisopropylethylamine (DIPEA) were purchased from Merck. 1-decanethiol and 4-hydroxybenzoic acid were purchased from Alfa Aesar. 3,5 dihydroxy benzoic acid was purchased from Apollo Scientific Ltd. Hexyl alcohol was purchased from ACROS Organics. Anhydrous potassium carbonate (K_2CO_3) was purchased from J. T. Baker. Anhydrous Na_2SO_4 (granular, 10-60 mesh) was purchased from Macron. Silica Gel 60Å, 0.040-0.063mm, Sodium Hydroxide and all solvents were purchased from Bio-Lab and were used as received. Deuterated solvents for NMR were purchased from Cambridge Isotope

Laboratories (CIL), Inc. Mini GeBAflex Tube were purchased from Gene Bio-Application Ltd. (GeBA) with a molecular weight cutoff of 8 kDa. Corning® 15 mL centrifuge tubes were purchased from Sigma Aldrich.

Synthesis

Synthesis of $mPEG_{5k}$-D-$(CX)_4$ amphiphilic hybrids:

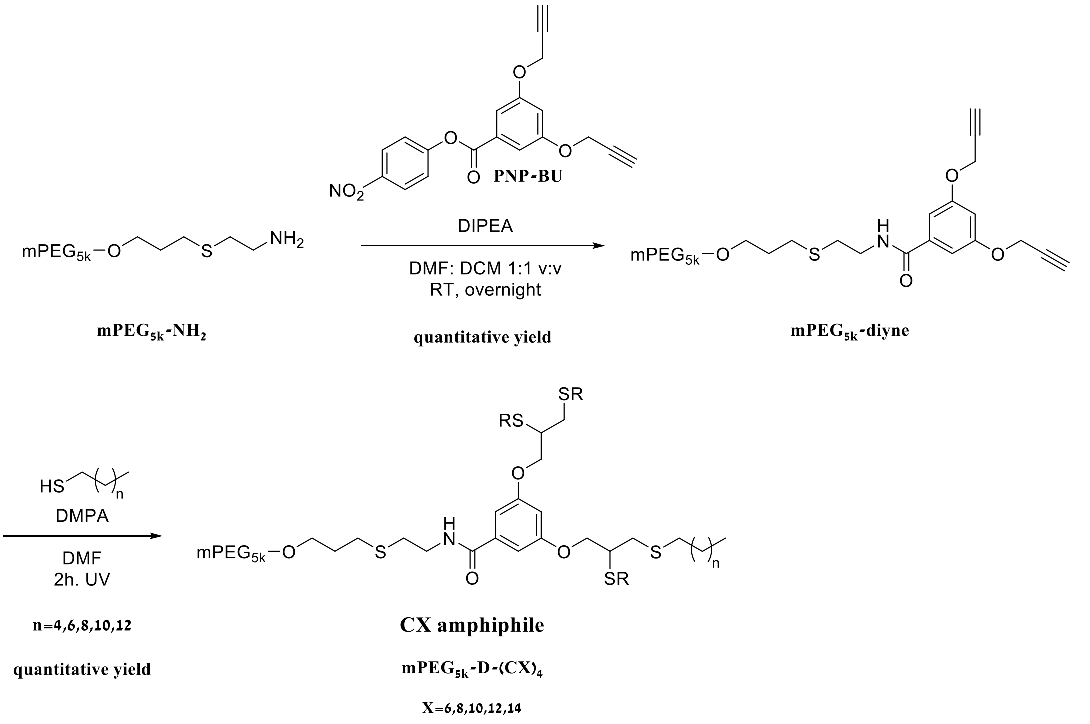

Figure S1 *- Synthetic Route for $mPEG_{5k}$-D-$(CX)_4$ amphiphilic hybrids.*

*$mPEG_{5k}$-NH_2 and $mPEG_{5k}$-diyne were synthesized as previously reported[1] and the spectroscopic characterization correlated well with these reports.

General procedure for thiol-yne reaction with of $mPEG_{5k}$-diyne with aliphatic thiols of various lengths:

$mPEG_{5k}$-diyne (1 eq), aliphatic thiol (40 eq) and DMPA (0.4 eq; 1 mol% with respect to the thiol) were dissolved in DMF (0.5 mL per 100 mg of hybrid). The solution was purged with nitrogen for 20 minutes and then stirred under UV light (365 nm) for 2 hours. Then, the reaction mixture was loaded as-is on a MeOH-based LH20 (Sephadex®) size exclusion column. Fractions that contained the product (identified by UV light and/or coloring with iodine) were unified, the organic solvents were evaporated to dryness and the white solid was dried under high vacuum.

mPEG$_{5k}$-D-(C6)$_4$: mPEG$_{5k}$-diyne (155 mg, 0.029 mmol), 1-hexanethiol (167 µL, 1.17 mmol) and DMPA (3 mg, 0.012 mmol) were reacted in DMF according to the general procedure. The product was obtained as a white solid in quantitative yield (160 mg).

^{1}H-NMR (400 MHz, Chloroform-*d*): δ 6.92 (d, J = 2.2 Hz, 2H, Ar-***H***), 6.63 (t, J = 5.7 Hz, 1H, -CH$_2$-N***H***-CO-Ar-), 6.59 (t, J =2.2 Hz, 1H, Ar-***H***), 4.29-4.07 (m, 4H, -C***H***$_2$-O-Ar), 3.80-3.43 (m, PEG backbone), 3.35 (s, 3H, C***H***$_3$-O-PEG), 3.09 (p, *J* = 6.0, 2H, -C***H***-S-), 2.99 – 2.80 (m, 4H, -CH$_2$-C***H***$_2$-S-), 2.74 (t, J = 6.5 Hz, 2H, -CH$_2$-C***H***$_2$-S-), 2.67 – 2.58 (m, 6H, -CH$_2$-C***H***$_2$-S-) 2.53 (t, *J* = 7.6 , 4H, -CH$_2$-C***H***$_2$-S-), 1.91 – 1.79 (m, 2H, -C***H***$_2$-CH$_2$-O-), 1.65 –1.48 (m, 8H, -C***H***$_2$-CH$_2$-S-),1.42-1.15 (m, 24H, -CH$_2$-C***H***$_2$-C***H***$_2$-CH$_2$- + -C***H***$_2$-CH$_3$), 0.88-0.83 (m, 12H, -CH$_2$-C***H***$_3$). ^{13}C-NMR (100 MHz, Chloroform-*d*): δ 167.1, 159.9, 136. 8, 106.0, 104.7, 70.7, 59.1, 45.2, 39.0, 34.8, 33.4, 32.0, 31.7, 31.5, 29.9, 29.8, 29.7, 28.3, 22.6, 14.1. SEC (DMF + 25 mM NH$_4$Ac): M_n = 5.0 kDa, Đ = 1.08. Expected M_n = 5.8 kDa. MALDI-TOF MS: molecular ion centered at 5.9 kDa.

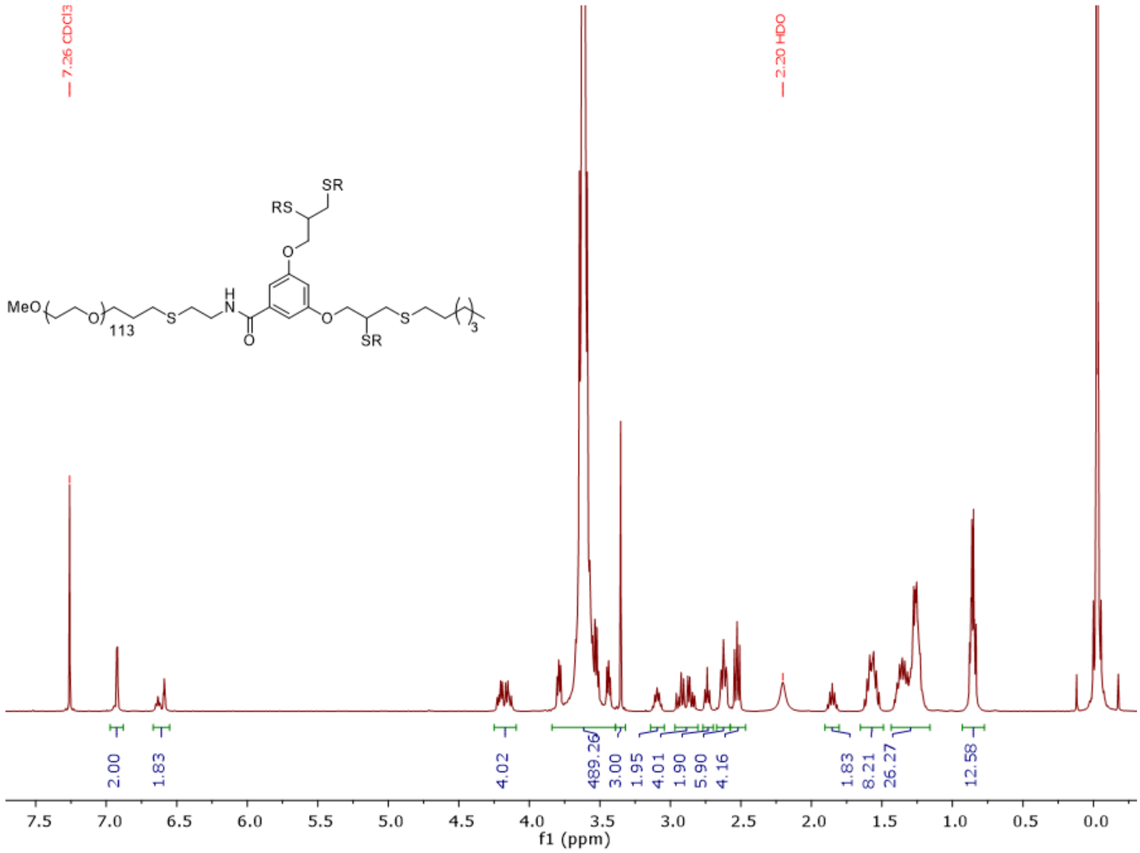

Figure S2– *^{1}H-NMR spectrum of compound mPEG$_{5k}$-D-(C6)$_4$ in CDCl$_3$.*

mPEG$_{5k}$-D-(C8)$_4$: mPEG$_{5k}$-diyne (160 mg, 0.030 mmol), 1-octanethiol (208 µL, 1.20 mmol) and DMPA (3 mg, 0.012 mmol) were reacted in DMF according to the general procedure. The product was obtained as a white solid in quantitative yield (160 mg).

^{1}H-NMR (400 MHz, Chloroform-*d*) δ 6.93 (d, J = 2.2 Hz, 2H, Ar-***H***), 6.63 (t, 1H, -CH$_2$-N***H***-CO-Ar-), 6.60 (t, J = 2.2 Hz, 1H, Ar-***H***), 4.37 – 4.06 (m, 4H, -C***H***$_2$-O-Ar), 3.81-3.43 (m, PEG backbone), 3.36 (s, 3H, C***H***$_3$-O-PEG), 3.10 (p, *J* = 6.0, 2H, -C***H***-S-), 2.99 – 2.82 (m, 4H, -CH$_2$-C***H***$_2$-S-), 2.75 (t, J = 6.5 Hz, 2H, -CH$_2$-C***H***$_2$-S-), 2.65-2.61 (m, 6H, -CH$_2$-C***H***$_2$-S-), 2.54 (t, *J* = 7.5, 4H, -CH$_2$-C***H***$_2$-S-), 1.86 (t, J = 6.7 Hz, 2H, -C***H***$_2$-CH$_2$-O-), 1.61 – 1.50 (m, 8H, -C***H***$_2$-CH$_2$-S-), 1.44 – 1.08 (m, 40H, -CH$_2$-C***H***$_2$-C***H***$_2$-CH$_2$- + -C***H***$_2$-CH$_3$), 0.88-0.84 (m, 12H, -CH$_2$-C***H***$_3$). ^{13}C-NMR (100 MHz, Chloroform-*d*): δ 167.1, 159.9, 136.8, 106.1, 104.4, 70.7, 59.1, 45.2, 39.0, 34.8, 33.4, 32.0, 31.8, 31.5, 29.9, 29.8, 29.7, 28.7, 28.6, 28.3, 22.6, 14.1. SEC (DMF + 25 mM NH_4Ac): M_n = 5.6 kDa, Đ = 1.04. Expected M_n = 5.9 kDa. MALDI-TOF MS: molecular ion centered at 6.0 kDa.

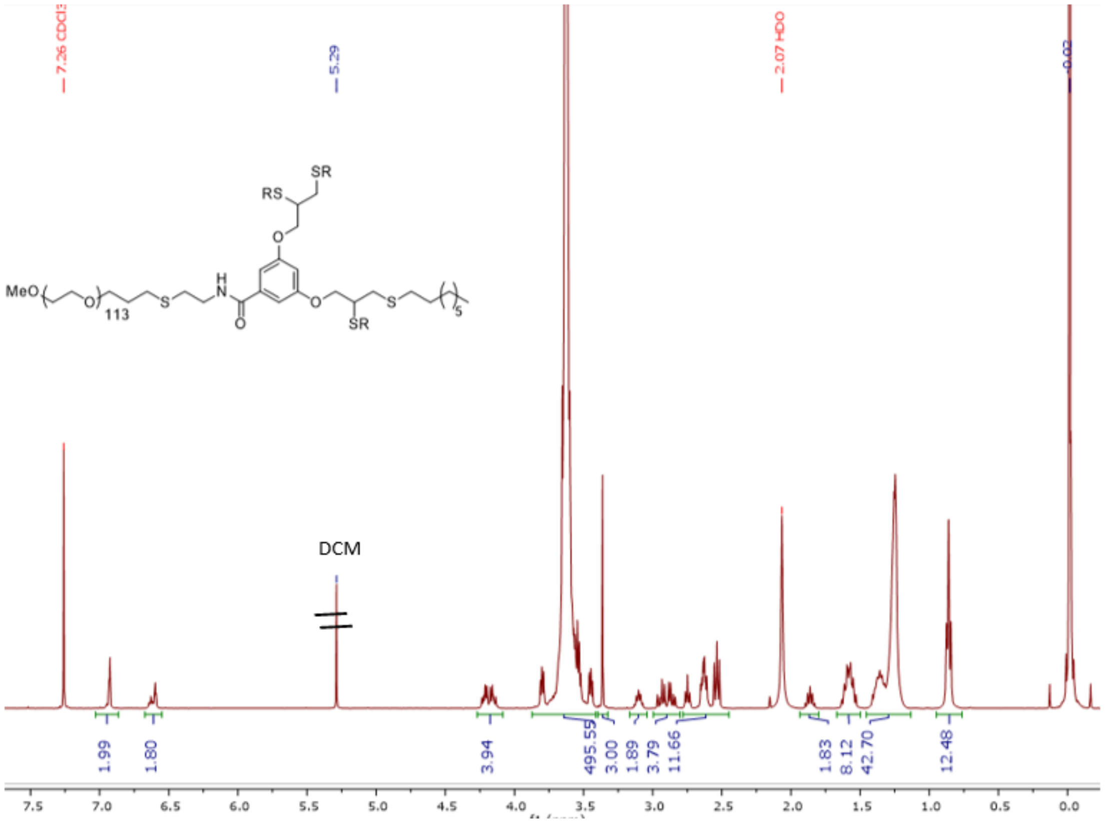

Figure S3 *– ^{1}H-NMR spectrum of compound mPEG$_{5k}$-D-(C8)$_4$ in $CDCl_3$.*

$mPEG_{5k}$-D-(C10)$_4$: $mPEG_{5k}$-diyne (103 mg, 0.019 mmol), 1-decanethiol (160 µL, 0.76 mmol) and DMPA (2 mg, 0.008 mmol) were reacted in DMF according to the general procedure. The product was obtained as a white solid in quantitative yield (114 mg).

^{1}H-NMR (400 MHz, Chloroform-*d*) δ 6.92 (d, *J* = 2.2 Hz, 2H, Ar-***H***), 6.63 (t, *J* = 5.8 Hz, 1H, -CH-N***H***-CO-Ar-), 6.59 (t, *J* = 2.2 Hz, 1H, Ar-***H***), 4.29 – 4.10 (m, 4H, -C$\boldsymbol{H}_2$-O-Ar), 3.81-3.44 (m, PEG backbone), 3.36 (s, 3H, C$\boldsymbol{H}_3$-O-PEG), 3.10 (p, *J* = 6.0, 2H, -C***H***-S-), 3.00 – 2.81 (m, 4H, -CH_2-C$\boldsymbol{H}_2$-S-), 2.74 (t, *J* = 6.5 Hz, 2H, -CH_2-C$\boldsymbol{H}_2$-S-), 2.65-2.60 (m, 6H, -CH_2-C$\boldsymbol{H}_2$-S-), 2.53 (t, *J* = 7.5, 4H, -CH_2-C$\boldsymbol{H}_2$-S-), 1.86 (p, *J* = 6.6 Hz, 2H, -C$\boldsymbol{H}_2$-CH_2-O-), 1.64 – 1.48 (m, 8H, -C$\boldsymbol{H}_2$-CH_2-S-), 1.39-1.24 (m, 56H, -CH_2-C$\boldsymbol{H}_2$-C$\boldsymbol{H}_2$-CH_2- + -C$\boldsymbol{H}_2$-CH_3), 0.86 (t, *J* = 6.7 Hz, 12H, -CH_2-C$\boldsymbol{H}_3$). ^{13}C-NMR (100 MHz, Chloroform-*d*): δ 167.1, 159.9, 136.2, 106.1, 104.8, 70.7, 59.2, 45.3, 39.0, 34.9, 33.5, 32.0, 30.0, 29.9, 29.7, 29.7, 29.5, 29.4, 28.4, 22.8, 14.3. SEC (DMF + 25 mM NH_4Ac): M_n=5.9 kDa, Đ = 1.05. Expected M_n = 6.0 kDa. MALDI-TOF MS: molecular ion centered at 6.1 kDa.

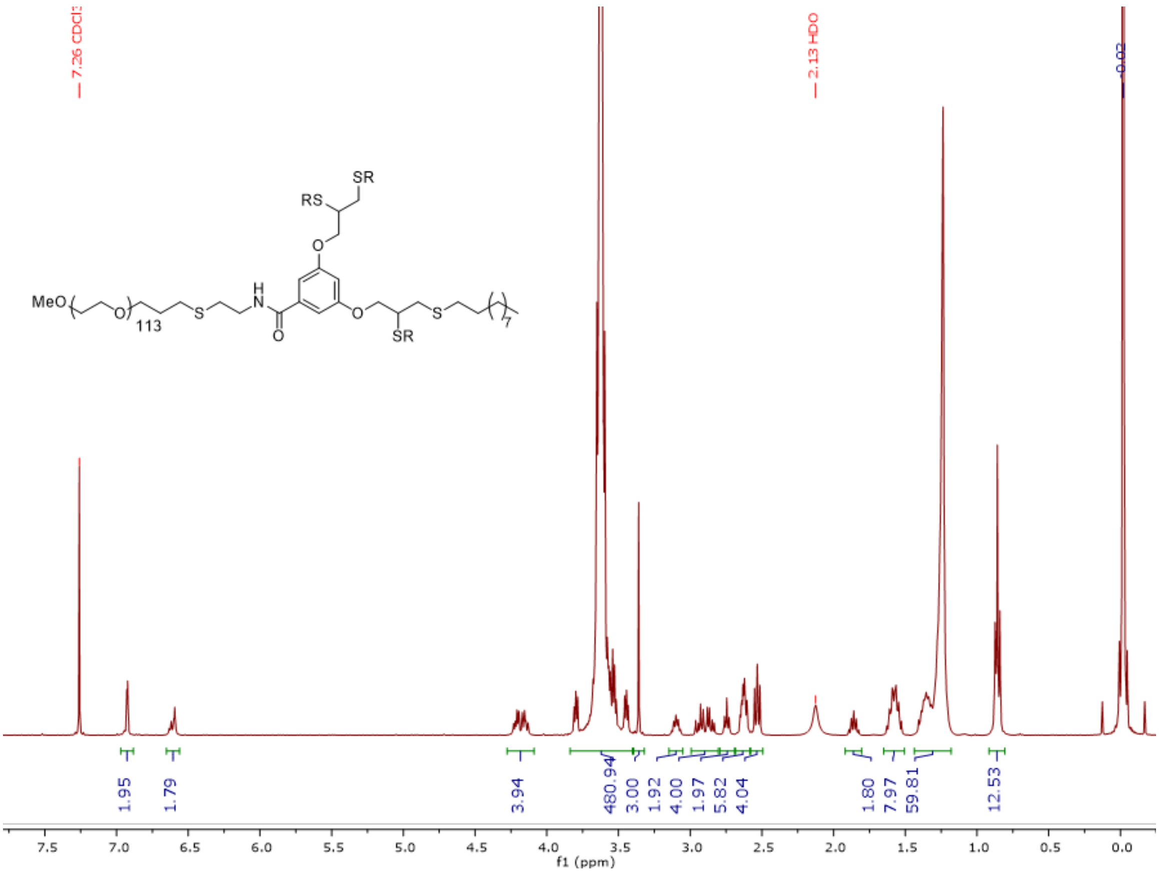

Figure S4 *– ^{1}H-NMR spectrum of compound $mPEG_{5k}$-D-(C10)$_4$ in $CDCl_3$.*

$mPEG_{5k}$-D-$(C12)_4$: $mPEG_{5k}$-diyne (157 mg, 0.029 mmol), 1-dodecanethiol (167 µL, 1.17 mmol) and DMPA (3 mg, 0.012 mmol) were reacted in DMF according to the general procedure. The product was obtained as a white solid in quantitative yield (173 mg).

^{1}H-NMR (400 MHz, Chloroform-*d*) δ 6.93 (d, *J* = 2.2 Hz, 2H, Ar-***H***), 6.62 (t, *J* = 5.8 Hz, 1H, -CH-N***H***-CO-Ar-), 6.60 (t, *J* = 2.2 Hz, 1H, Ar-***H***), 4.25 – 4.11 (m, 4H, -C$\boldsymbol{H}_2$-O-Ar), 3.81-3.44 (m, PEG backbone), 3.36 (s, 3H, C$\boldsymbol{H}_3$-O-PEG), 3.10 (p, *J* = 6.0 Hz , 2H, -C***H***-S-), 3.00 – 2.81 (m, 4H, -CH_2-C$\boldsymbol{H}_2$-S-), 2.75 (t, *J* = 6.5 Hz, 2H, -CH_2-C$\boldsymbol{H}_2$-S-), 2.65-2.60 (m, 6H, -CH_2-C$\boldsymbol{H}_2$-S-), 2.53 (t, *J* = 7.4 Hz, 4H, -CH_2-C$\boldsymbol{H}_2$-S-), 1.86 (p, *J* = 6.6 Hz, 2H, -C$\boldsymbol{H}_2$-CH_2-O-), 1.63-1.53 (m, 8H, -C$\boldsymbol{H}_2$-CH_2-S-), 1.37-1.18 (m, 72H, -CH_2-C$\boldsymbol{H}_2$-C$\boldsymbol{H}_2$-CH_2- + -C$\boldsymbol{H}_2$-CH_3), 0.86 (t, *J* = 6.7 Hz, 12H, -CH_2-C$\boldsymbol{H}_3$). ^{13}C-NMR (100 MHz, Chloroform-*d*): δ 167.1, 159.9, 136.8, 106.1, 104.8, 70.7, 59.1, 45.3, 39.1, 34.9, 33.5, 32.0, 31.8, 30.0, 29.9, 29. 8, 29.8, 29.7, 29.7, 29.5, 29.4, 28.4, 22.8, 14.2. SEC (DMF + 25 mM NH_4Ac): M_n = 6.1 kDa, Đ = 1.05. Expected M_n = 6.2 kDa. MALDI-TOF MS: molecular ion centered at 6.3 kDa.

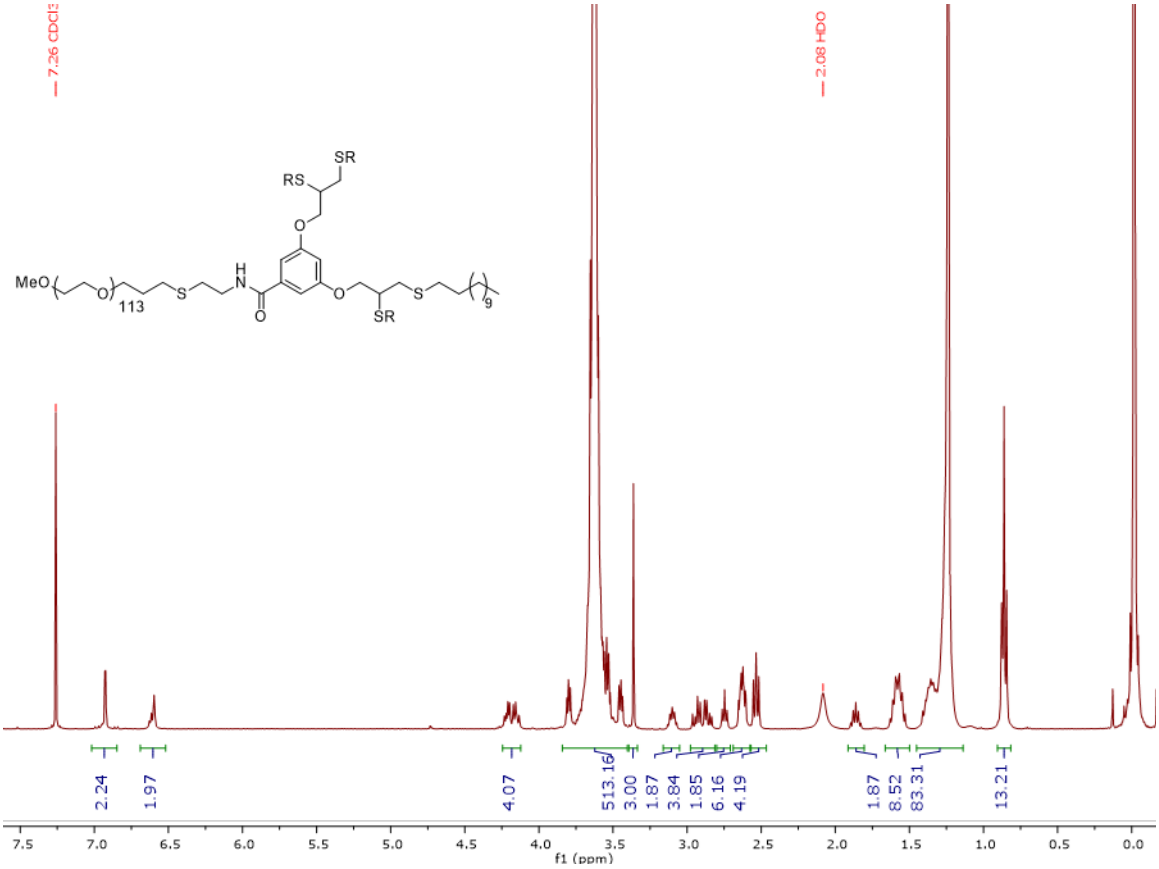

Figure S5– *^{1}H-NMR spectrum of compound $mPEG_{5k}$-D-$(C12)_4$ in $CDCl_3$.*

<u>mPEG$_{5k}$-D-(C14)$_4$:</u> mPEG$_{5k}$-diyne (153 mg, 0.029 mmol), 1-tetradecanethiol (316 µL, 1.16 mmol) and DMPA (3 mg, 0.012 mmol) were reacted in DMF according to the general procedure. The product was obtained as a white solid in quantitative yield (170 mg).

^{1}H-NMR (400 MHz, Chloroform-*d*) δ 6.92 (d, *J* = 2.2 Hz, 2H, Ar-***H***), 6.62 (t, *J* = 5.8 Hz, 1H, -CH-N***H***-CO-Ar-), 6.59 (t, *J* = 2.2 Hz, 1H, Ar-***H***), 4.27 – 4.10 (m, 4H, -C***H***$_2$-O-Ar), 3.80-3.43 (m, PEG backbone), 3.35 (s, 3H, C***H***$_3$-O-PEG), 3.09 (p, *J* = 6.0, 2H, -C***H***-S-), 2.98 – 2.81 (m, 4H, -CH$_2$-C***H***$_2$-S-), 2.74 (t, *J* = 6.5 Hz, 2H, -CH$_2$-C***H***$_2$-S-), 2.64-2.62 (m, , 6H, -CH$_2$-C***H***$_2$-S-), 2.53 (t, *J* = 7.4 Hz, 4H, -CH$_2$-C***H***$_2$-S-), 1.94 – 1.78 (m, 2H, -C***H***$_2$-CH$_2$-O-), 1.67 – 1.50 (m, 8H, -C***H***$_2$-CH$_2$-S-), 1.41 – 1.07 (m, 88H, -CH$_2$-C***H***$_2$-C***H***$_2$-CH$_2$- + -C***H***$_2$-CH$_3$), 0.85 (t, *J* = 6.7, 12H, -CH$_2$-C***H***$_3$). ^{13}C-NMR (100 MHz, Chloroform-*d*): δ 167.1, 159.9, 136.8, 106.0, 104.7, 70.7, 59.1, 45.2, 39.0, 34.8, 33.4, 32.0, 31.8, 30.0, 29. 8, 29.7, 29.6, 29.5, 29.4, 28.3, 22. 8, 14.2. SEC (DMF + 25 mM NH$_4$Ac): M_n = 6.2 kDa, Đ = 1.05. Expected M_n = 6.3 kDa. MALDI-TOF MS: molecular ion centered at 6.4 kDa.

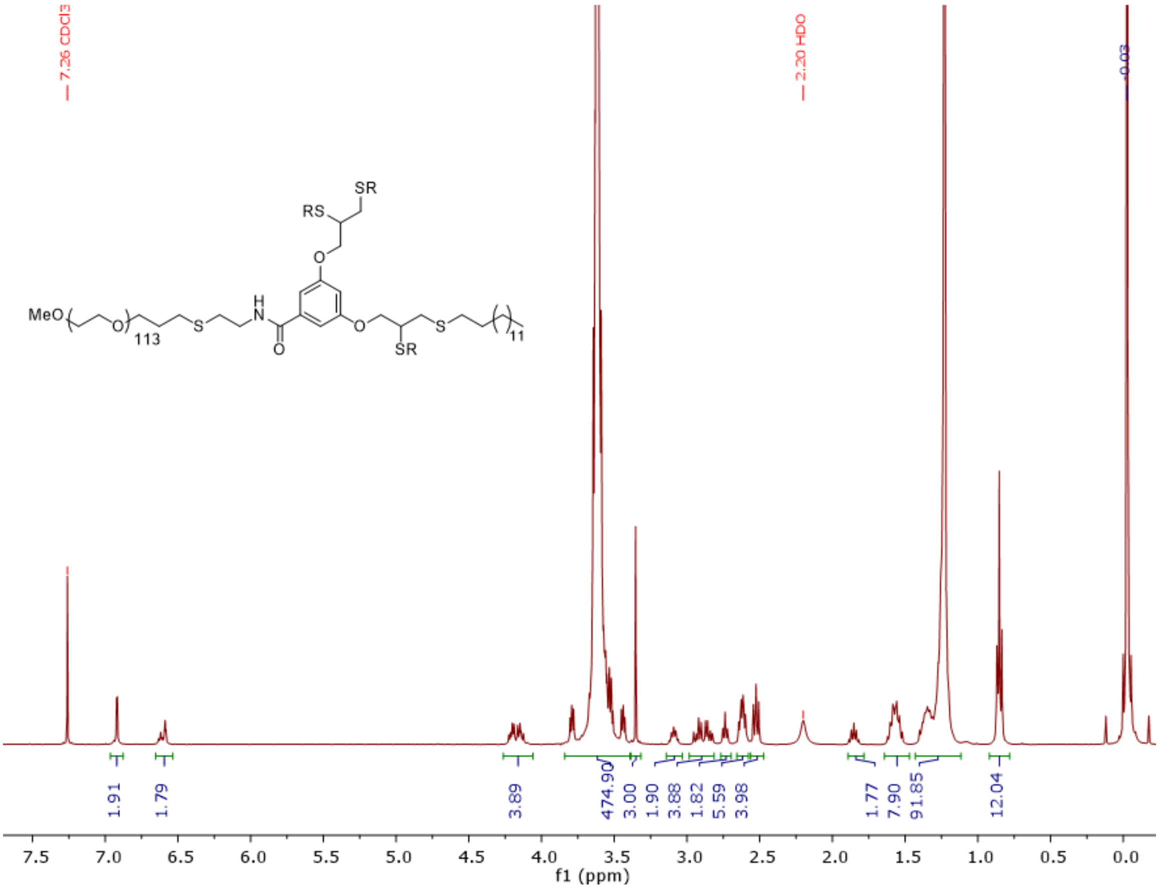

Figure S6– *^{1}H-NMR spectrum of compound mPEG$_{5k}$-D-(C14)$_4$ in CDCl$_3$.*

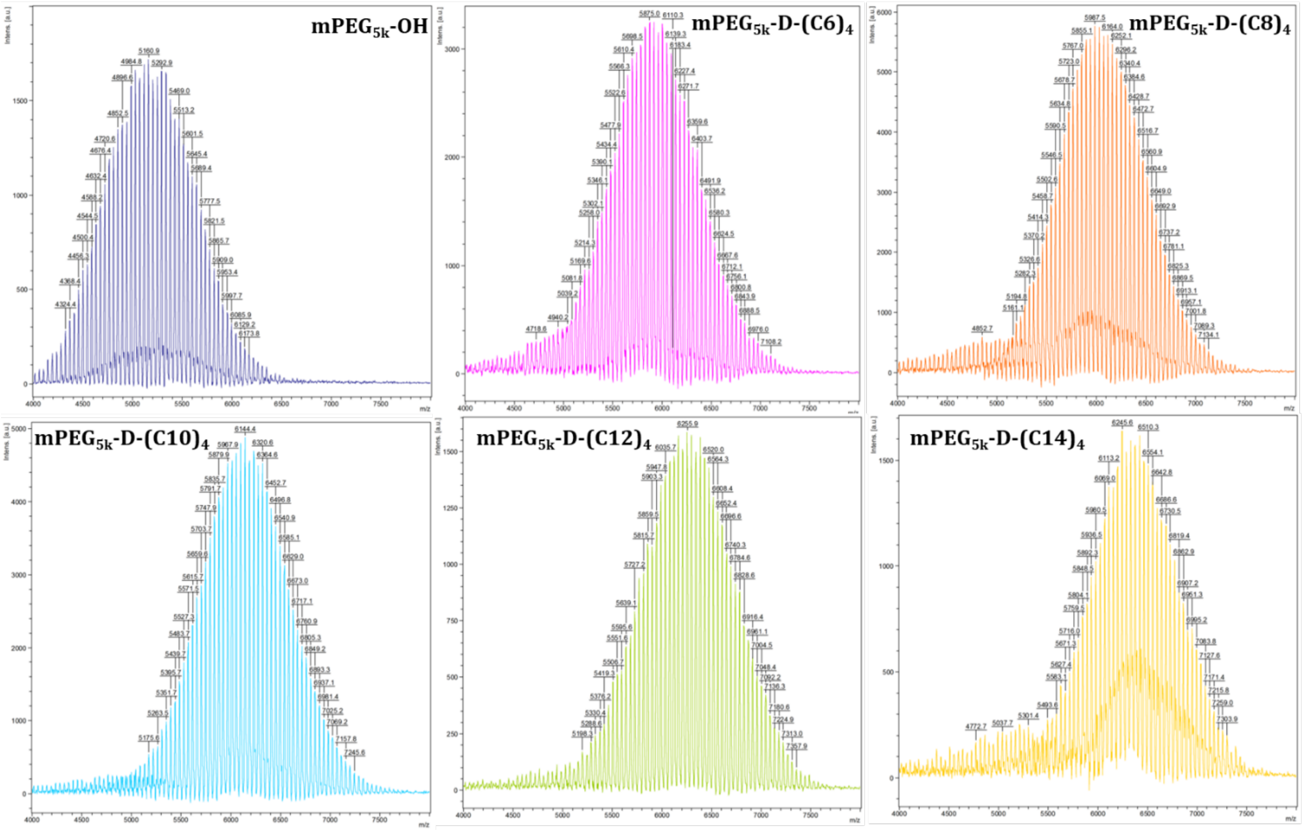

Figure S7 *- MALDI spectra of mPEG*$_{5k}$*-D-(CX)*$_4$ *hybrids.*

Synthesis of Propargylated substrates:

4-nitrophenol propargyl ether (PNPPE substrate):

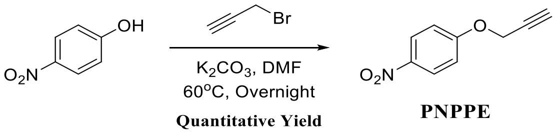

Figure S8*– Synthesis of PNP-propargyl ether.*

PNPPE: 4-nitrophenol (1.0 g, 7 mmol, 1 eq) was dissolved in DMF (10 mL). Anhydrous K_2CO_3 (1.1 g, 13 mmol, 1.8 eq) and 80% propargyl bromide in toluene (2.4 mL, 21 mmol, 3 eq) were added and the flask was brought to reflux and stirred overnight. The flask cooled to RT, then the reaction mixture was poured into ice-cold water (50 mL) and stirred for 1 hour. The precipitate was collected by filtration and washed with water. The product was obtained as an off-white solid in quantitative yield (1.3 g).

^{1}H-NMR (400 MHz, Chloroform-d): δ 8.23 (d, J = 9.3 Hz, 2H, Ar-***H***), 7.06 (d, J = 9.3 Hz, 2H, Ar-***H***), 4.80 (d, J = 2.4 Hz, 2H, -O-C***H***$_2$-C≡CH), 2.59 (t, J = 2.5 Hz, 1H, -C≡C***H***). ^{13}C-NMR (100 MHz, Chloroform-*d*): δ 162.4, 142.2, 125.9, 115.1, 77.2, 76.8, 56.4. MS calculated for $C_9H_7NO_3$ 178.17 (MH^+), found 178.20.

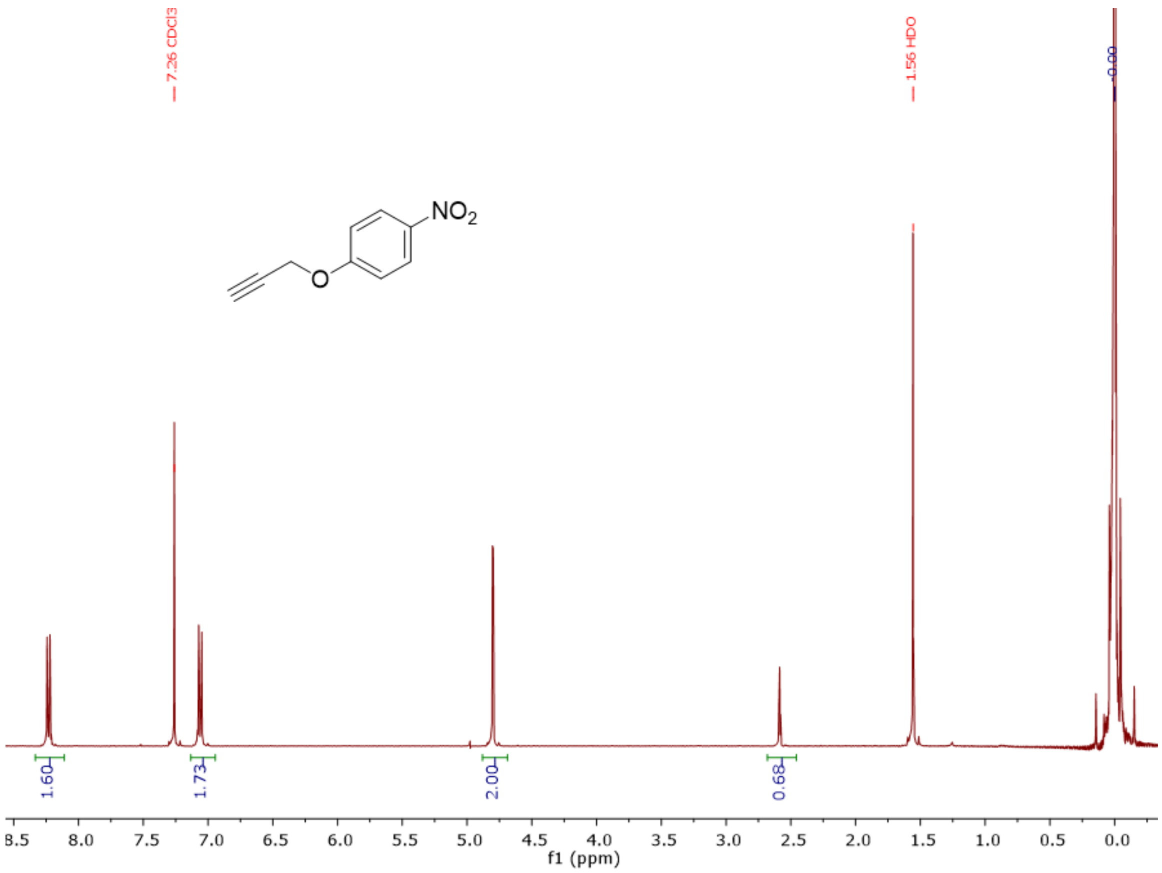

Figure S9 *– ^{1}H-NMR spectrum of compound PNP-propargyl ether in $CDCl_3$.*

<u>Synthesis of 4-(propargyloxy) benzoic acid alkyl ester:</u>

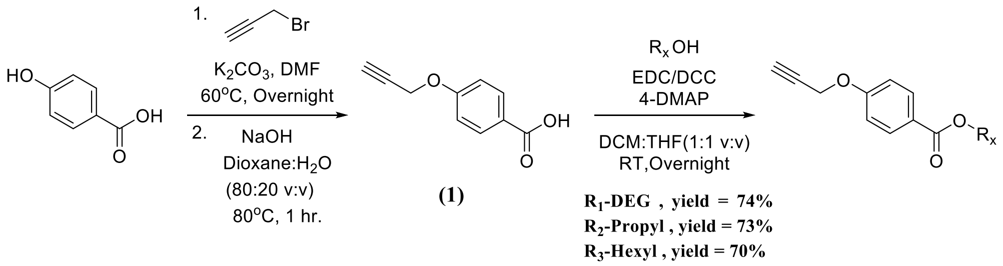

Figure S10*- Synthetic Route for 4-(propargyloxy) benzoic acid alkyl ester.*

<u>4-(propargyloxy) benzoic acid (1):</u> 4-hydroxybenzoic acid (3.0 g, 22 mmol, 1 eq) was dissolved in DMF (40 mL). Anhydrous K_2CO_3 (9.0 g, 66 mmol, 3 eq) and 80% propargyl bromide in toluene (7.3 mL, 66 mmol, 3 eq) were added and the flask was heated to 60°C and stirred overnight. Flask was cooled to RT, then diluted with EA (100 mL) and washed with water and brine (2x100 mL) and the organic solvents were removed in vacuum. The obtained residue was re-dissolved in dioxane: H_2O 80:20 v/v (45 mL), NaOH 4 N (11 mL, 43 mmol, 2 eq) was added and the flask was heated to 80°C for 1 hour. The reaction cooled to RT and the pH was acidified to pH=1~2 using HCl 1N to afford white precipitate,

followed by the addition of 100 mL water. The solid was filtered, washed with water and re-dissolved in EA. The combined organic phase was dried under Na_2SO_4, filtered and the EA was evaporated. The solid was dried under high vacuum. The product was obtained as a white solid in 60% yield (2.3 g).

^{1}H-NMR (400 MHz, DMSO-d$_6$): δ 7.90 (d, J = 8.9 Hz, 2H, Ar-***H***), 7.06 (d, J = 8.9 Hz, 2H, Ar-***H***), 4.88 (d, J = 2.4 Hz, 2H, -O-C***H***$_2$-C≡CH), 3.61 (t, J = 2.4 Hz, 1H, -C≡C***H***). ^{13}C-NMR (DMSO-d$_6$): δ 167.0, 160.8, 131.3, 123.8, 114.7, 78.8,78.7, 55.7. MS calculated for $C_9H_7NO_3$ (negative mode) 175.16 (m/z, [M-H]$^-$), found 175.20.

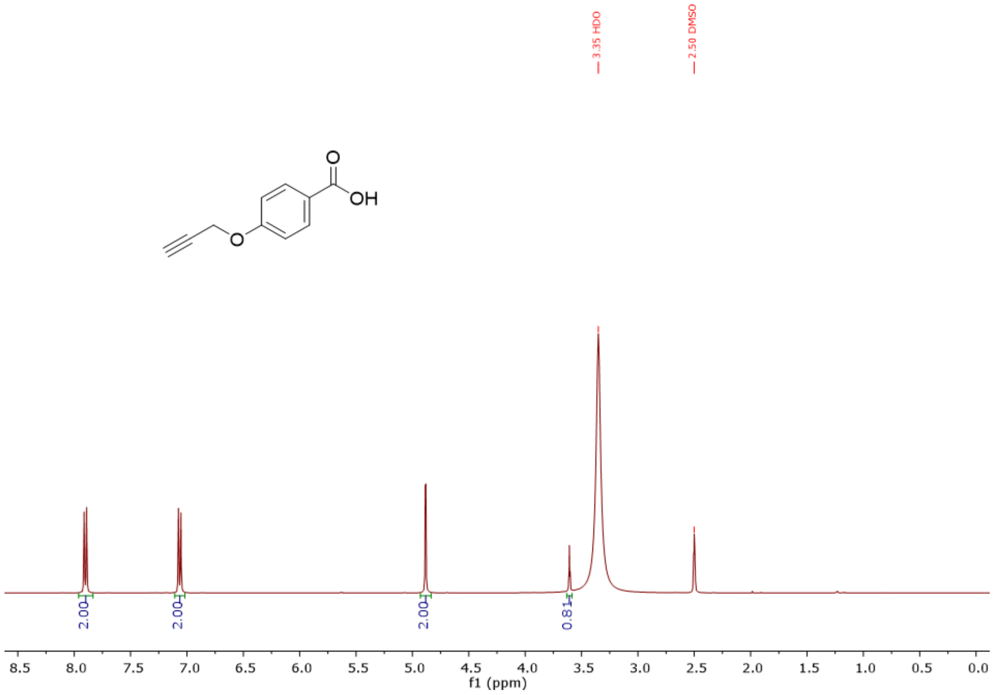

Figure S11- *^{1}H-NMR spectrum of compound 4-(propargyloxy) benzoic acid in DMSO-d$_6$.*

4-(propargyloxy) benzoic acid diethylene glycol ester: 4-(propargyloxy) benzoic acid (125 mg, 0.7 mmol, 1 eq), 2-(2-methoxyethoxy)ethanol (DEG, 0.1 mL, 0.85 mmol, 1.2 eq), EDC (163 mg, 0.85 mmol, 1.2 eq) and 4-DMAP (17 mg, 0.1 mmol, 0.2 eq) were dissolved in a mixture on DCM:THF 1:1 v:v (3 mL) and stirred overnight. Organic solvents were evaporated to dryness and the product was purified using flash silica chromatography (Hex: EA, 60:40). Product was obtained as a viscous liquid in 74% yield (117 mg).

^{1}H-NMR (400 MHz, Chloroform-d): δ 8.03 (d, J = 8.9 Hz, 2H, Ar-***H***), 7.00 (d, J = 8.9 Hz, 2H, Ar-***H***), 4.75 (d, J = 2.4 Hz, 2H, -O-C***H***$_2$-C≡CH), 4.46 (m, 2H, -C***H***$_2$-O-CO$_2$), 3.83 (m, 2H, -C***H***$_2$-O), 3.69 (m, 2H, -C***H***$_2$-O), 3.57 (m, 2H, -C***H***$_2$-O), 3.39 (s, 3H, C***H***$_3$-O), 2.54 (t, J = 2.4 Hz, 1H, -C≡C***H***). ^{13}C-NMR (100 MHz, Chloroform-*d*): δ 166.2, 161.3, 131.8, 123.5, 114.6, 77.9, 76.2, 72.0, 70.7, 69.4, , 64.0, 59.1, 55.9. MS calculated for $C_{15}H_{18}O_5$ 279.31 (MH$^+$), found 279.30.

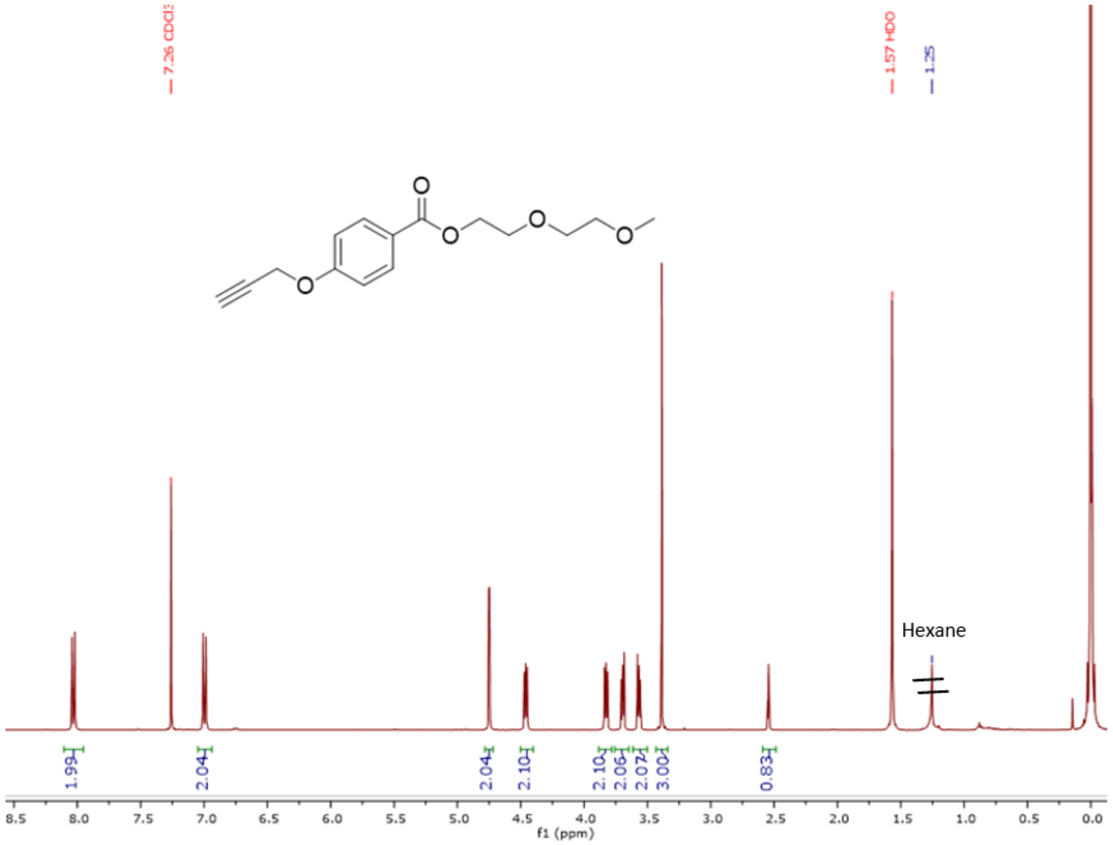

Figure S12- *^{1}H-NMR spectrum of compound 4-(propargyloxy) benzoic acid diethylene glycol ester in $CDCl_3$.*

4-(propargyloxy) benzoic acid propyl ester: 4-(propargyloxy) benzoic acid (500 mg, 2.8 mmol, 1 eq), 1-propanol (0.43 mL, 5.6 mmol, 2 eq), DCC (1.17 g, 5.6 mmol, 2 eq) and 4-DMAP (104 mg, 0.8 mmol, 0.3 eq) were dissolved in a mixture on DCM:THF 1:1v:v (4 mL) and stirred overnight. The reaction was filtered through celite, and the celite was washed with DCM. Organic solvents were evaporated to dryness. Product was purified using flash silica chromatography (Hex: EA, 90:10). Product was obtained as a viscous oil in 73% yield (450 mg).

^{1}H-NMR (400 MHz, Chloroform-d): δ 8.02 (d, J = 8.9 Hz, 2H, Ar-***H***), 7.00 (d, J = 8.9 Hz, 2H, Ar-***H***), 4.75 (d, J = 2.4 Hz, 2H, -O-C***H***$_2$-C≡CH), 4.26 (t, J = 6.6 Hz, 2H, -C***H***$_2$-CO$_2$-), 2.54 (t, J = 2.4 Hz, 1H, -C≡C***H***), 1.78 (m, 2H, -C***H***$_2$-CH$_2$- CO$_2$-), 1.03 (t, J = 7.4 Hz, 3H, C***H***$_3$-CH$_2$). ^{13}C-NMR (100 MHz, Chloroform-*d*): δ 166.4, 161.2, 131.6, 124.0, 114.6, 78.0, 76.2, 66.5, 56.0, 22.3, 10.7. MS calculated for $C_{12}H_{12}O_3$ 219.24 (MH$^+$), found 219.30.

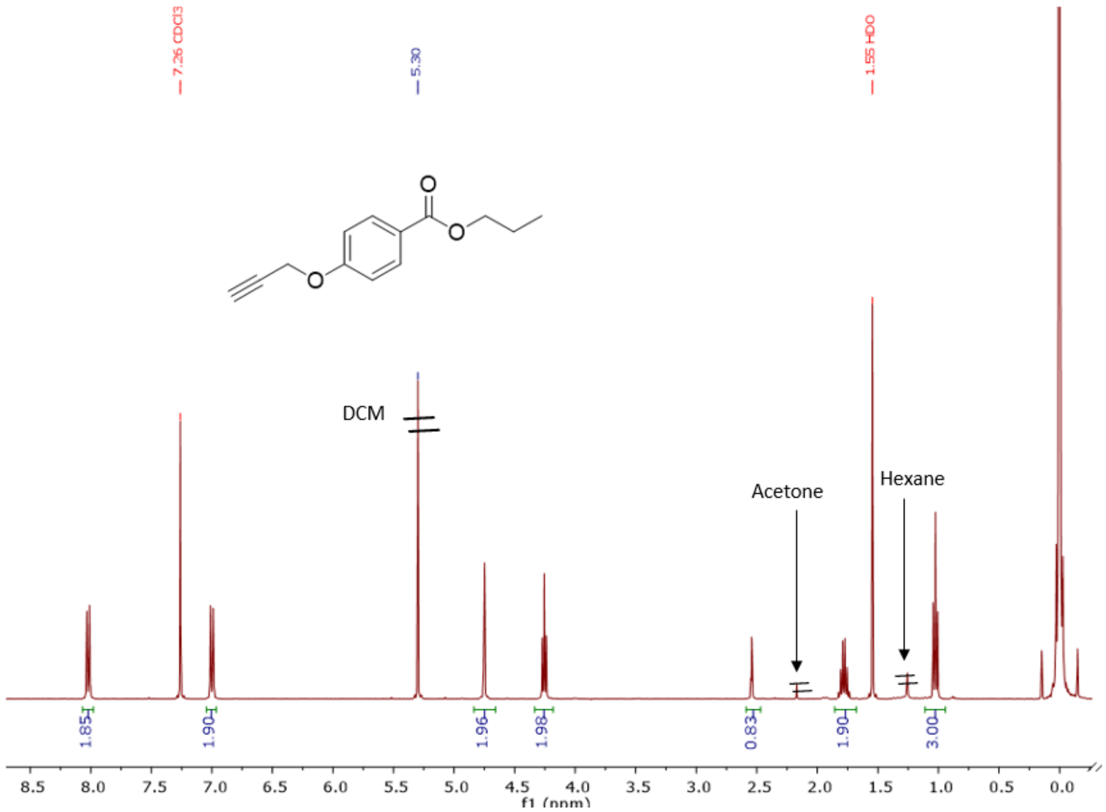

***Figure S13**- ^{1}H-NMR spectrum of compound 4-(propargyloxy) benzoic acid propyl ester in $CDCl_3$.*

4-(propargyloxy) benzoic acid hexyl ester: 4-(propargyloxy) benzoic acid (163 mg, 0.9 mmol, 1 eq), 1-hexanol (0.35 mL, 2.8 mmol, 3 eq), DCC (573 mg, 2.8 mmol, 3 eq) and 4-DMAP (34 mg, 0.3 mmol, 0.3 eq) were dissolved in a mixture on DMC: THF 1:1 v:v (3 mL) and stirred overnight. The reaction was filtered through celite, and the celite was washed with DCM. Organic solvents were evaporated to dryness. Product was purified using flash silica chromatography (Hex: EA 95:5). Product was obtained as a white solid in 70% yield (169 mg).

^{1}H-NMR (400 MHz, Chloroform-d): δ 8.01 (d, J = 9.0 Hz, 2H, Ar-***H***), 7.00 (d, J = 8.9 Hz, 2H, Ar-***H***), 4.75 (d, J = 2.4 Hz, 2H, -O-C***H***$_2$-C≡CH), 4.28 (t, J = 6.7 Hz, 2H, -C***H***$_2$-CO$_2$-), 2.54 (t, J = 2.4 Hz, 1H, -C≡C***H***), 1.75 (m, 2H, -C***H***$_2$-CH$_2$- CO$_2$-), 1.44 (m, 2H, -C***H***$_2$-CH$_2$-) , 1.34 (m, 4H, -C***H***$_2$-CH$_2$-), 0.91 (t, 3H, C***H***$_3$-CH$_2$). ^{13}C-NMR (100 MHz, Chloroform-*d*): δ 166.4, 161.2, 136.8, 124.0, 114.6, 78.0, 76.2, 65.1, 56.0, 31.6, 28.9, 25.9, 22.7, 14.1. MS calculated for $C_{15}H_{18}O_5$ 261.34 (MH$^+$), found 261.30.

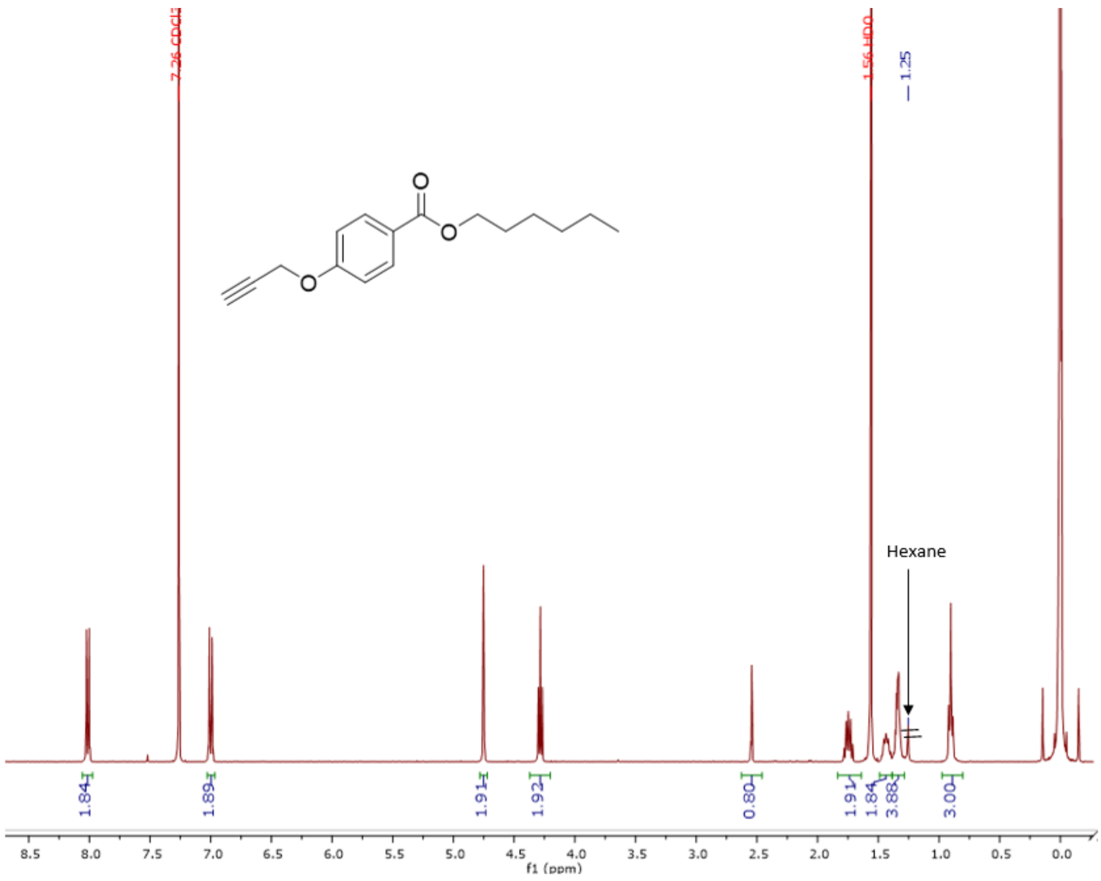

Figure S14- *^{1}H-NMR spectrum of compound 4-(propargyloxy) benzoic acid hexyl ester in $CDCl_3$.*

Characterization of PEG-dendron hybrids
HPLC measurements

Instrument: Waters Alliance e2695

Column: Aeris WIDEPORE, C4, 3.6 μm, 150x4.6 mm

Column temperature: 30°C

Sample temperature: 37°C

Solution A: 0.1% $HClO_4$:ACN 95:5 v/v

Solution B: 0.1% $HClO_4$:ACN 5:95 v/v

Solution C: ACN

Flow rate: 1 mL/min

Gradient program for 15 minutes injection:

Time [minutes]	Sol. A [%]	Sol. B [%]	Sol. C [%]
0.0	95	0	5
1.0	95	0	5
8.0	0	95	5
10.0	0	95	5
10.1	95	0	5
15.0	95	0	5

Injection volume: 30 μL

Seal wash: H_2O:MeOH 90:10 v/v

Needle wash: MeOH

Detector: Waters 2998 photodiode array detector

Sampling rate: 2 points/sec

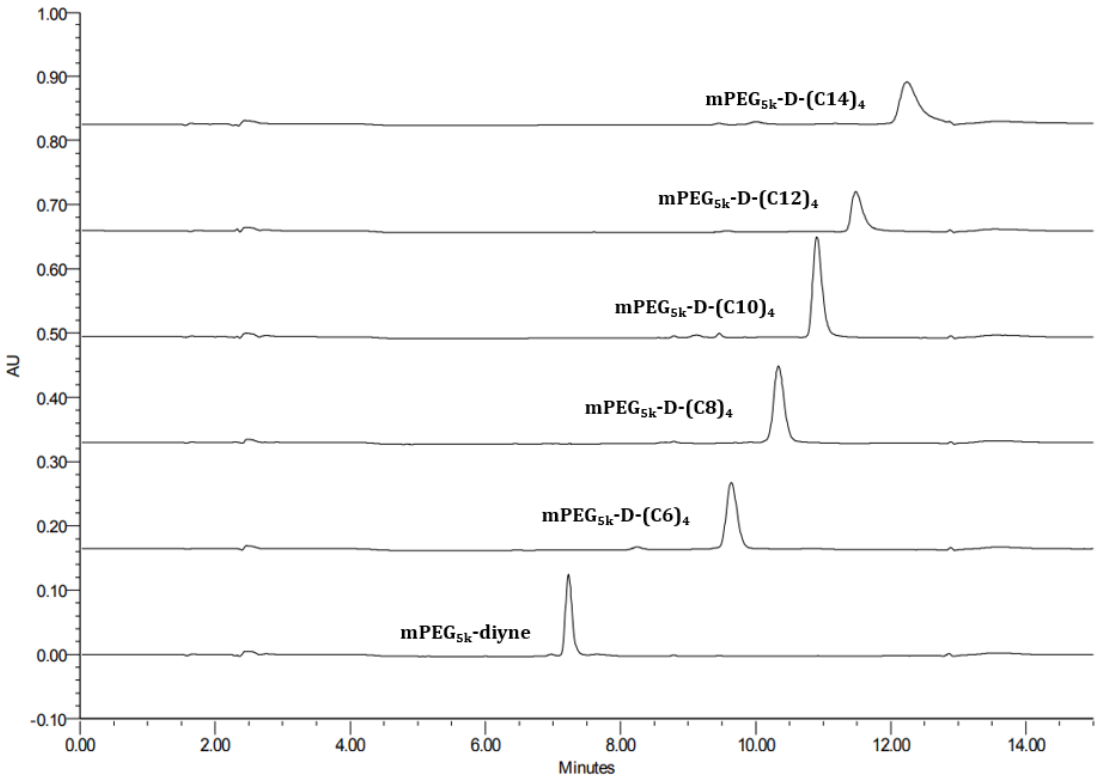

Figure S15 *– HPLC chromatogram overlay of mPEG$_{5k}$-diyne and mPEG$_{5k}$-D-(CX)4 hybrids.*

Size exclusion chromatography (SEC)

Instrument method:

Instrument: Malvern Viscotek GPCmax

Columns: 2xPSS GRAM 1000Å + PSS GRAM 30Å

Column temperature: 50°C

Flow rate: 0.5 mL/min

Injection time: 90 min

Injection volume: 50 µL from a 10 mg/mL sample

Diluent + mobile phase: DMF + 25mM NH_4Ac

Needle wash: DMF

Detector: Viscotek VE3580 RI detector

Sample preparation: The amphiphiles were directly dissolved in the diluent to give a final concentration of 10 mg/mL and filtered with 0.45 µm PTFE syringe filter.

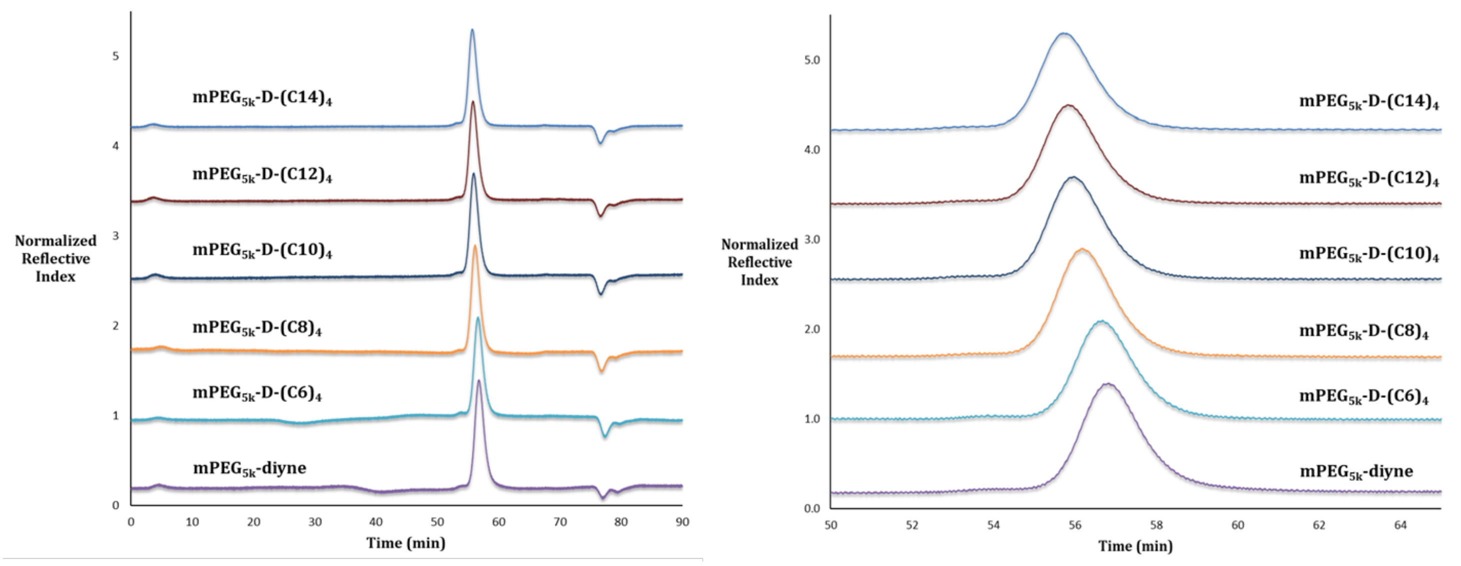

Figure S16 *– SEC traces overlay of mPEG$_{5k}$-diyne and mPEG$_{5k}$-D-(CX)$_4$ amphiphiles. Both complete and zoomed in chromatograms are displayed.*

Characterization of PEG-dendron micellar structures
Critical micelles' concentration (CMC)

General procedure of measurement:

Preparation of diluent:

Nile Red stock solution (0.88 mg/mL in ethanol) was diluted into a phosphate buffer saline (137 mM NaCl, 10 mM phosphate, 2.7 mM KCl; pH 7.4) to afford a final concentration of 1.25 µM.

Preparation and measurement of samples:

The PEG-dendron amphiphiles were directly dissolved in the diluent to give a final concentration of 500 µM. Solution was vortexed vigorously until the amphiphile completely dissolved and further sonicated for 15 minutes in an ultrasonic bath. The solutions were consecutively diluted by a factor of 1.5 with the diluent to afford a series of 24 samples for each amphiphile. 150 µL of each sample was loaded onto a 96 well plate and a fluorescence emission scan was performed for each well. To determine the hybrid's CMC – the maximum emission of Nile Red (at about 630 nm) was plotted as a function of the amphiphile's concentration. This procedure was repeated thrice for each amphiphile, and mean value is reported as the CMC value and the standard deviation as measurement error.

Instrument method:

Instrument: TECAN Infinite M200Pro

Excitation: 550 nm

Emission intensity scan: 580-800 nm

Step: 2 nm

Number of flashes: 15

Gain: 100

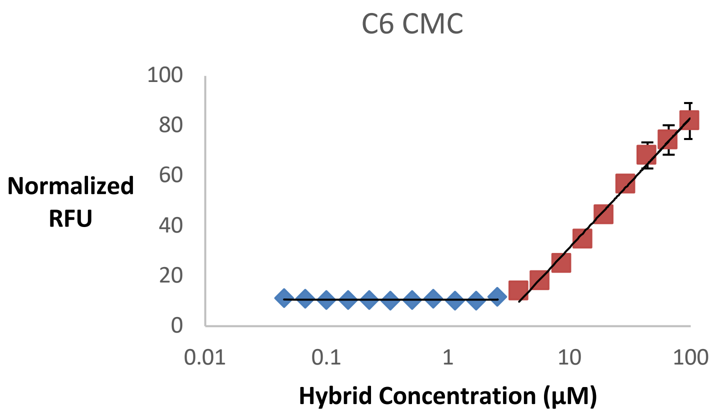

Figure S17- *CMC measurement of C6 amphiphile (mPEG$_{5k}$-D-(C6)$_4$).*

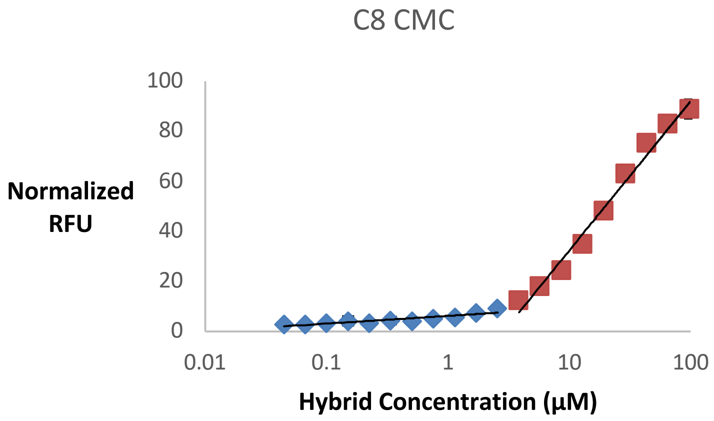

Figure S18- *CMC measurement of C8 amphiphile (mPEG$_{5k}$ -D-(C8)$_4$).*

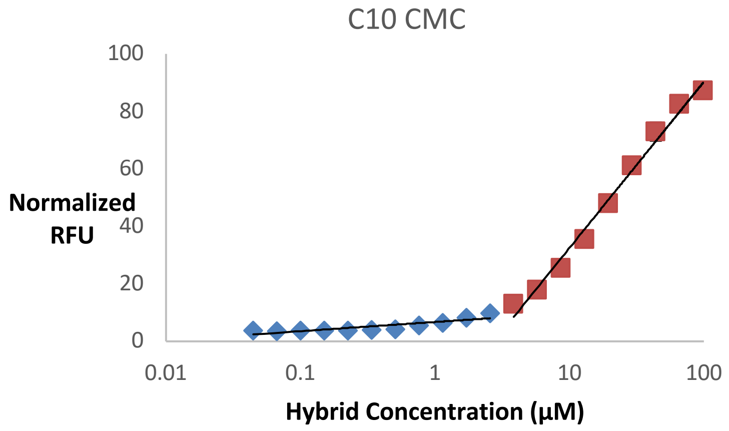

Figure S19- *CMC measurement of C10 amphiphile (mPEG$_{5k}$ -D-(C10)$_4$).*

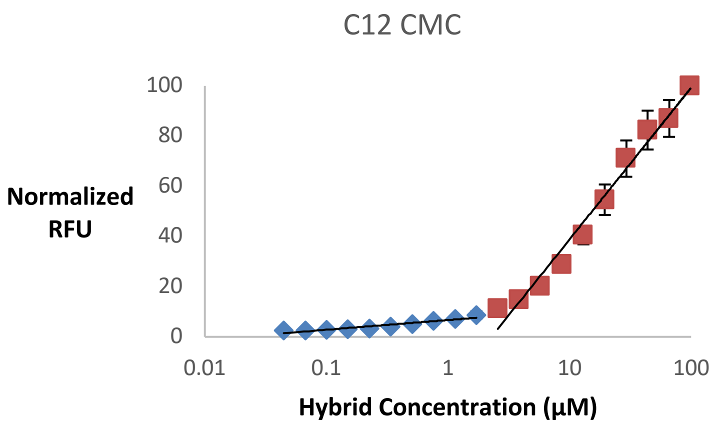

***Figure S20**- CMC measurement of C12 amphiphile (mPEG$_{5k}$ -D-(C12)$_4$).*

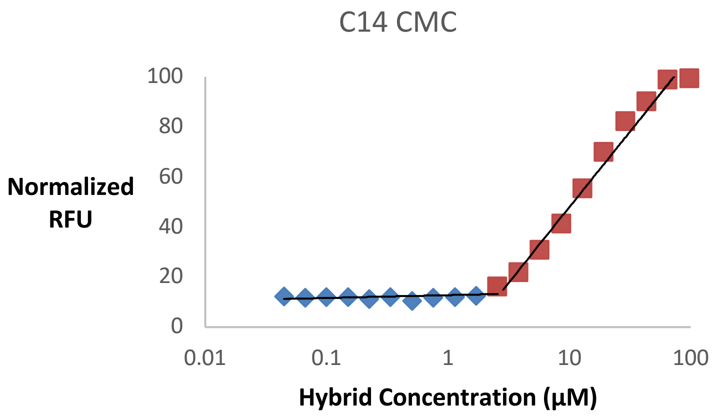

***Figure S21**- CMC measurement of C14 amphiphile (mPEG$_{5k}$ -D-(C14)$_4$).*

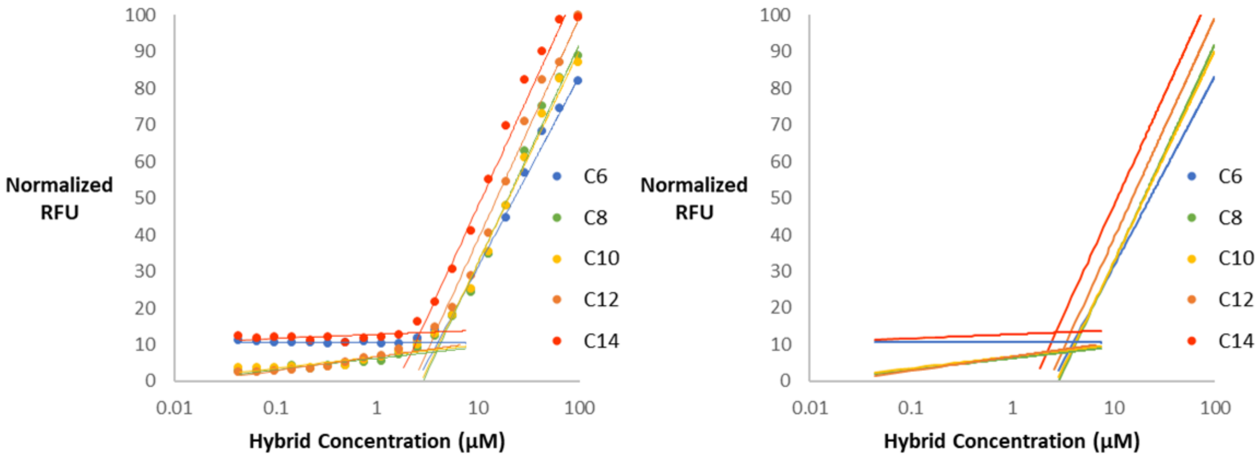

***Figure S22**- Overlay of CMC measurements of CX amphiphiles.*

Dynamic light scattering

Sample preparation:

Detailed sample preparation method is described under the "General protocol for the preparation of palladium-embedded micellar nanoreactors and depropargylation experiments setup". The amount of $Pd(OAc)_2$ solution was modified accordingly to yield the desire amphiphile:metal molar ratio.

A

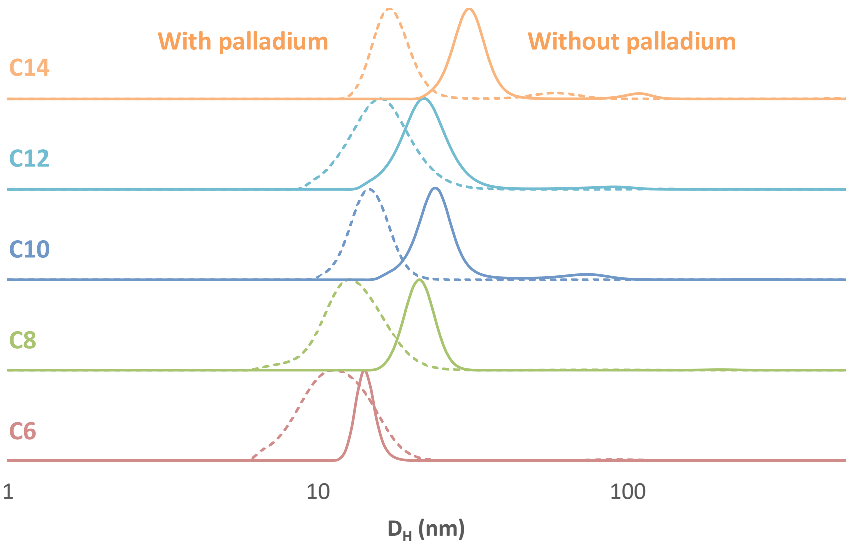

B

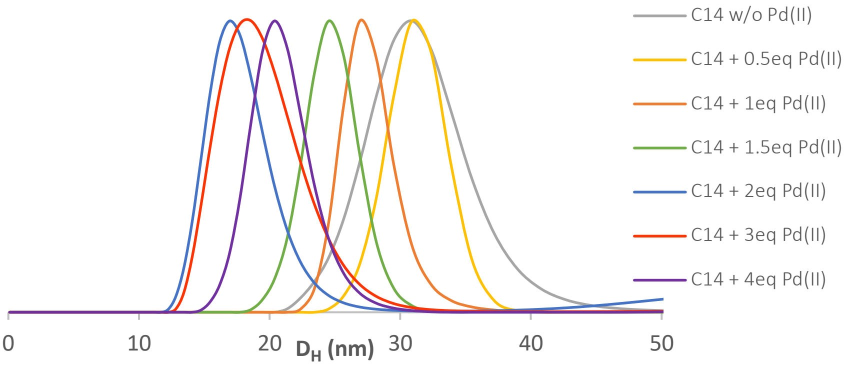

Pd(OAc)₂ eq.	***D_H (nm)***
0	*31±4*
0.5	*31±3*
1	*28±3*
1.5	*25±2*
2	*18 ±3*
3	*19 ±3*
4	*21 ±3*

Figure S23 *– **A)** DLS size measurements overlay of CX amphiphiles; X=6,8,10,12,14, with (full line) and without (dashed line) the presence of $Pd(OAc)_2$ salt. [Amphiphile] = 42 μM; [$Pd(OAc)_2$] = 83 μM. **B)** DLS size measurements overlay and analyzed D_H values of C14 amphiphiles with increasing amount of $Pd(OAc)_2$ salt. [Amphiphile] = 42 μM.*

TEM imaging

Sample preparation:

The amphiphiles solution with or without Pd salt were prepared as described for the depropargylation experiments. 30 μL of the solution were dropped onto carbon coated copper grids. The excessive solvent of the droplet was wiped away using a filter paper and the sample grids were left to dry in air at RT. Then, grids were inspected in transmission electron microscope (TEM), operated at 80 kV (JEM-1400Plus).

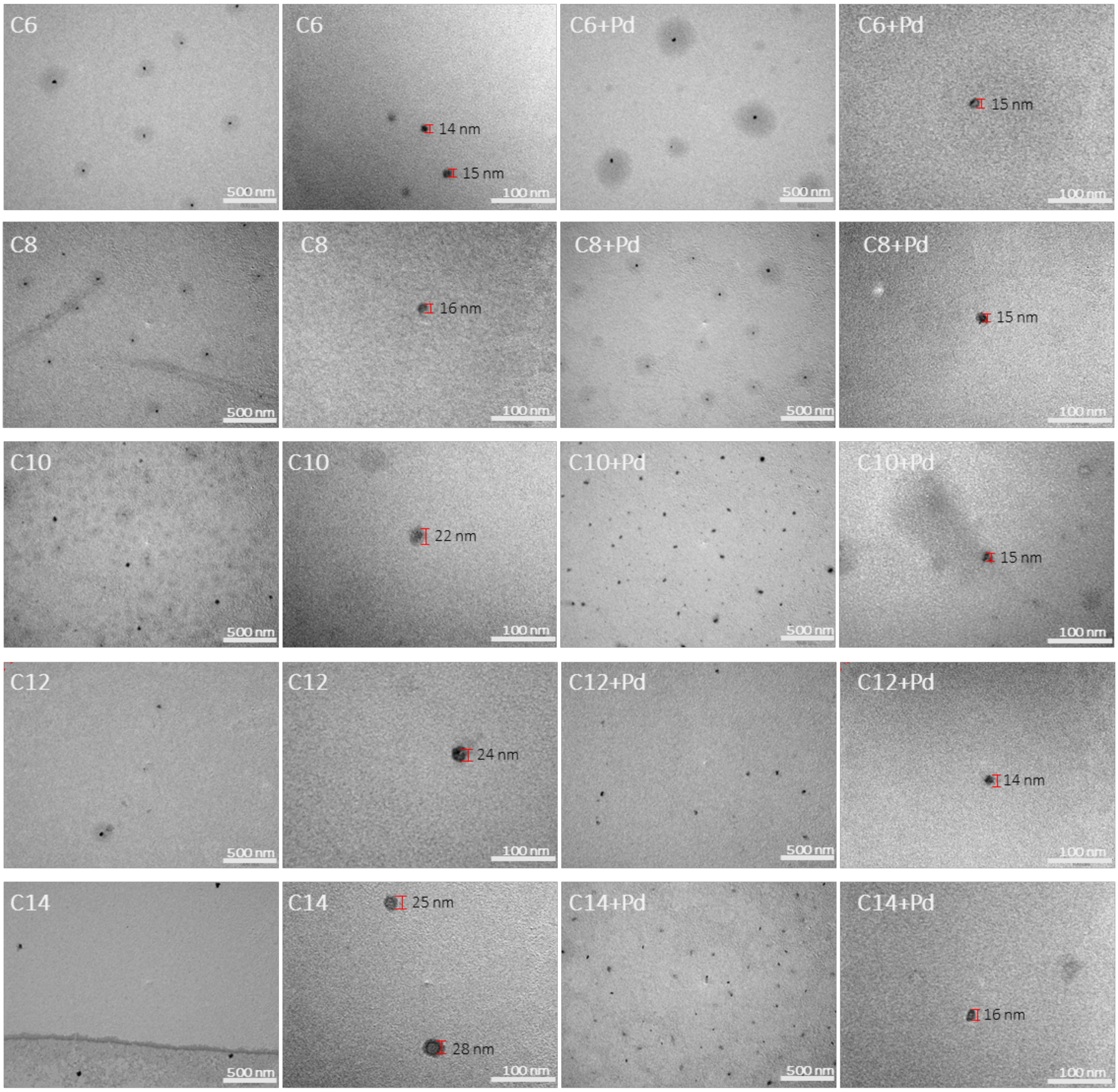

Figure S24 *– TEM images of CX micelles; X=6,8,10,12,14, with (right columns) and without (left columns) the presence of palladium acetate salt.*

Evaluation of complex formation

A series of solutions with different molar ratios of metal to amphiphile, ranging between 0% to 200%, were prepared and the complex formation was monitored by ^{1}H-NMR. Each component was separately dissolved in chloroform-*d* to give a final concentration of 3.2 mM. Different volumes from each solution were combined to provide the desired molar fraction, stirred briefly and measured immediately after sample perpetration.

An overlay of the normalized spectra is presented in figure S25, with the assigned peaks divided into two groups; purple – the peak's integral has changed significantly upon metal addition, green – no significant change was observed.

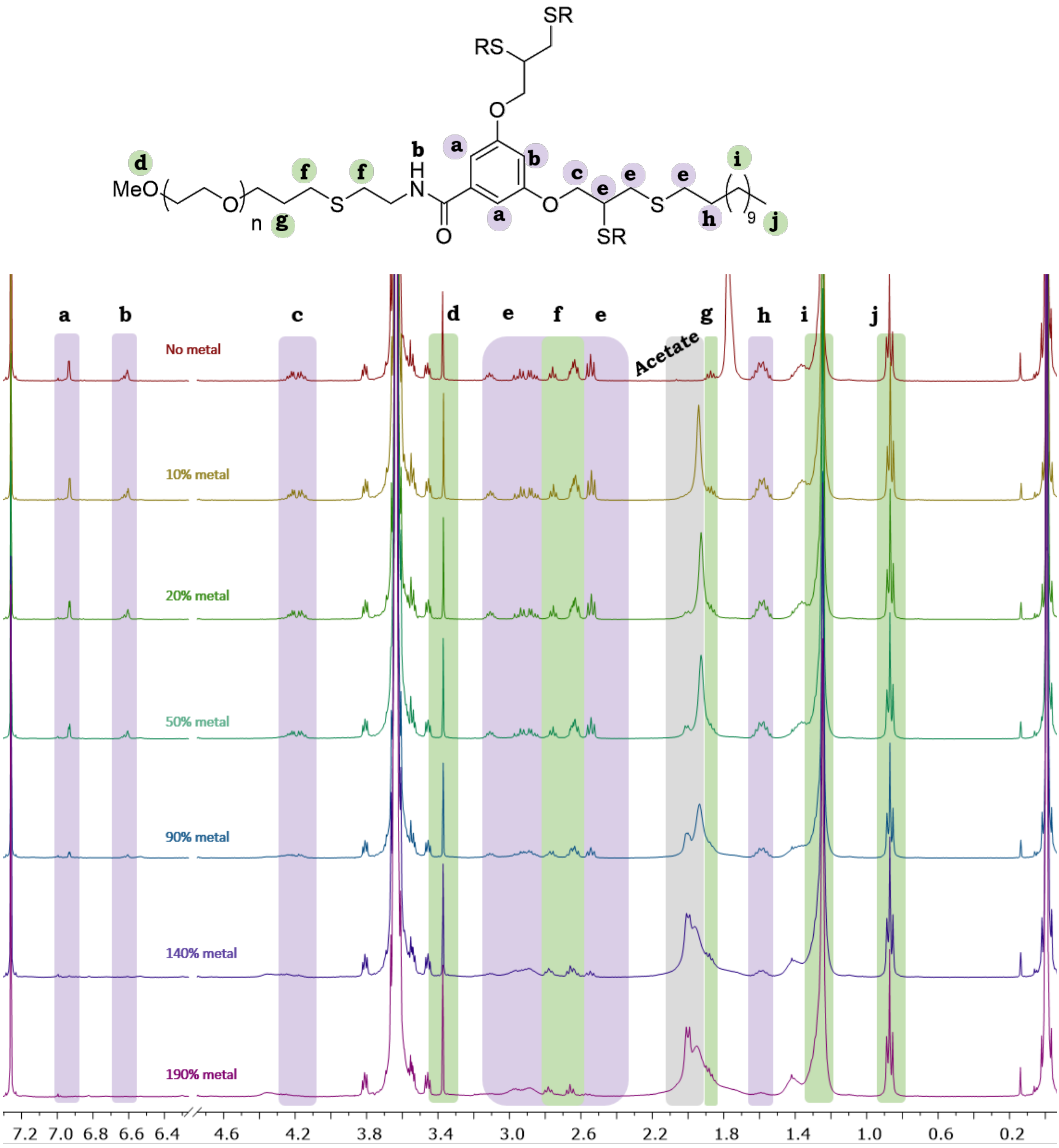

Figure S25 - *^{1}H-NMR overlay spectra of mPEG$_{5k}$-D-(C12)$_4$ in CDCl$_3$ in the presence of increasing metal ratios.*

Depropargylation experiments

General protocol for the preparation of palladium-embedded micellar nanoreactors and depropargylation experiments setup

Stock solutions of $Pd(OAc)_2$ and CX amphiphiles were made separately in acetone at a concentration of 166 μM and 83 μM, respectively. The solutions were mixed at a ratio of 1:1 v/v and vortexed briefly. The organic solvent was removed, and the mixture was dried under high vacuum. Then, the mixture was re-dissolved in phosphate buffer saline (PBS, pH=7.4) to form the micellar nanoreactor, with final concentrations of 83 μM and 42 μM for the metal salt and the amphiphile, respectively.

Stock solutions of the substrates were made separately by directly dissolving the solids in DMSO to give a final concentration of 22.3 mM for PNPPE substrate, and 13.8 mM for PropylBPE, HexylPBe and DEGBPE substrates.

To initiate the reaction, the substrates were added to the aqueous nanoreactor solution (7.5 μL of substrate solution per 1 mL) and the vials were vortexed briefly. The catalysis was followed using HPLC at 37°C, by monitoring the area under the peak of the substrate at the maximal wavelength. This procedure was repeated thrice for each set of amphiphile and substrate. Samples of amphiphiles and substrates in the absence of palladium were used as a control for monitoring the stability of the substrate solution over time and to ensure its hydrolysis cannot be catalyzed by the micellar system alone. A control of substrates and metal in the absence of the micellar structures could not be measured since both compounds have poor water solubility.

Table S1. Propargyl containing substrates – properties and kinetic analysis.

Entry	Substrate	Substrate LogP#	Product LogP#	Amphiphile	k (h^{-1})	$t_{1/2}$ calc. (h)	$t_{1/2}$ exp. (h)
1	PNPPE	2.1	1.4	no amphiphile*	0.077	9.0	7.5
2				C6	0.054	12.9	12.7
3				C8	0.061	11.4	11.5
4				C10	0.067	10.4	10.2
5				C12	0.078	8.9	8.5
6				C14	0.091	7.6	7.5
7	PropylBPE	2.8	2.3	no amphiphile*	0.094	7.4	6.2
8				C6	0.27	2.5	2.8
9				C8	0.33	2.1	2.5
10				C10	0.35	2.0	2.3
11				C12	0.40	1.7	2.1
12				C14	0.42	1.6	2.0
13	DEGBPE	1.6	1.2	no amphiphile*	0.12	5.7	5.7
14				C6	0.035	20.0	18.0
15				C14	0.055	12.7	11.0
16	HexylBPE	4.0	3.5	no amphiphile*	0.082	8.5	7.9
17				C6	1.9	0.4	0.7
18				C14	3.0	0.2	0.3

Table S1. LogP values and kinetic parameters (reaction rate, k, calculated and experimental $t_{1/2}$ values) for the different propargyl containing substrates. # = Calculated via ChemDraw Version 18.2 * = PBS:Acetone mixture (1:1, v/v).

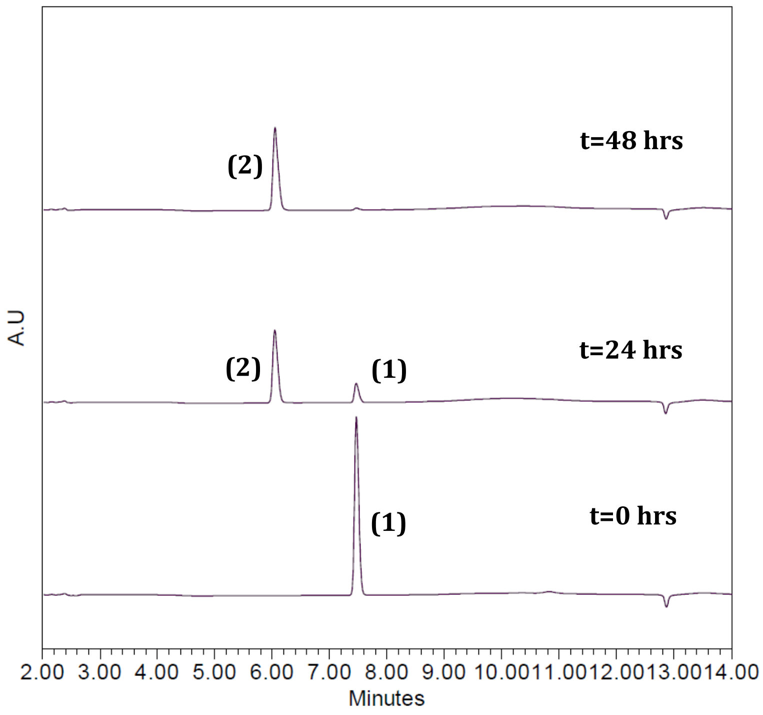

Figure S26- *Representative HPLC chromatogram overlay (taken at 307 nm), showing the transformation of PNPPE (1) to PNP (2) in the presence of Pd(OAc)$_2$ loaded micelles made of mPEG$_{5k}$-D-(C14)$_4$ amphiphiles.*

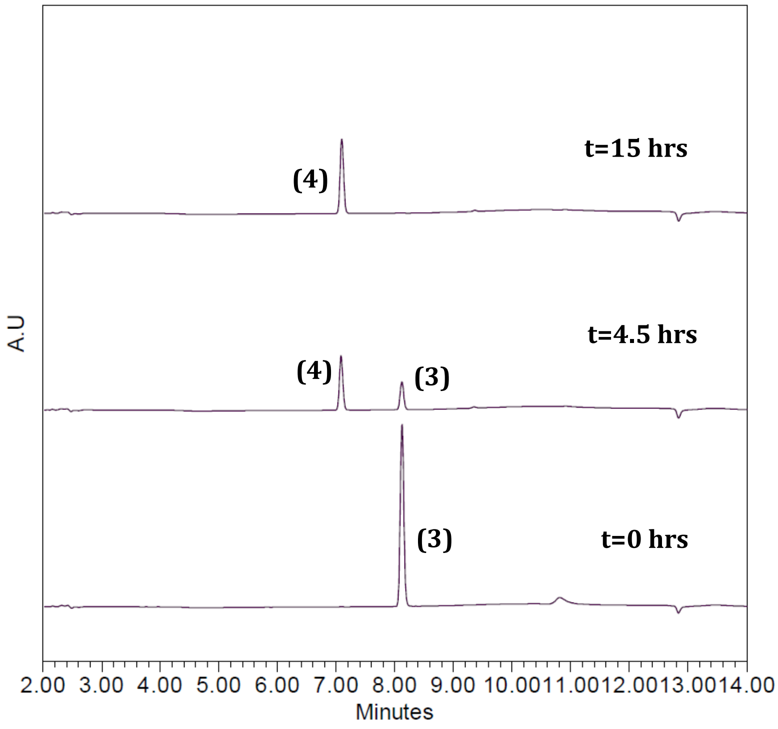

Figure S27- *Representative HPLC chromatogram overlay (taken at 252 nm), showing the transformation of PropylBPE substrate (3) to its' de-propargylated product (4) in the presence of Pd(OAc)$_2$ loaded micelles made of mPEG$_{5k}$-D-(C14)$_4$ amphiphiles.*

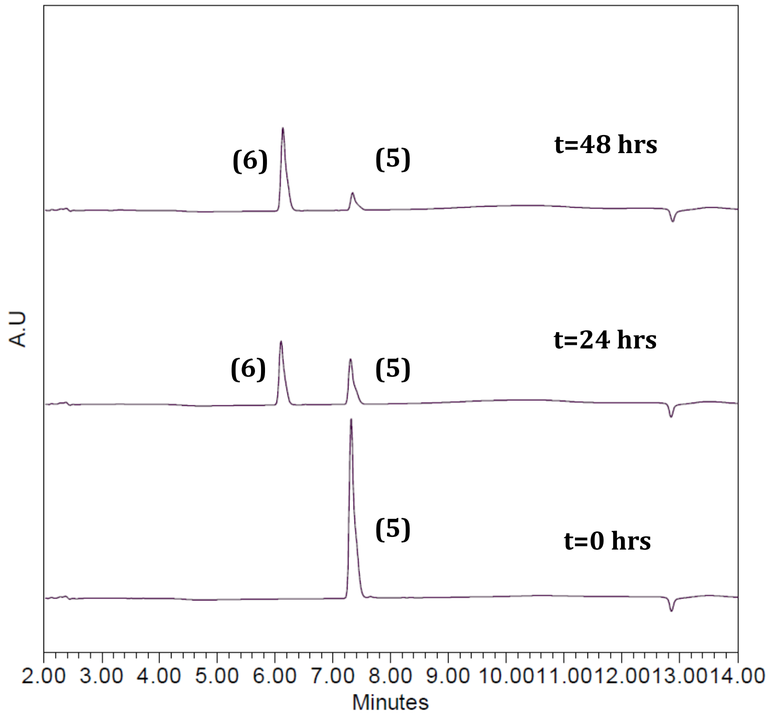

Figure S28- *Representative HPLC chromatogram overlay (taken at 252 nm), showing the transformation of DEGBPE substrate (5) to its' de-propargylated product (6) in the presence of Pd(OAc)$_2$ loaded micelles made of mPEG$_{5k}$-D-(C14)$_4$ amphiphiles.*

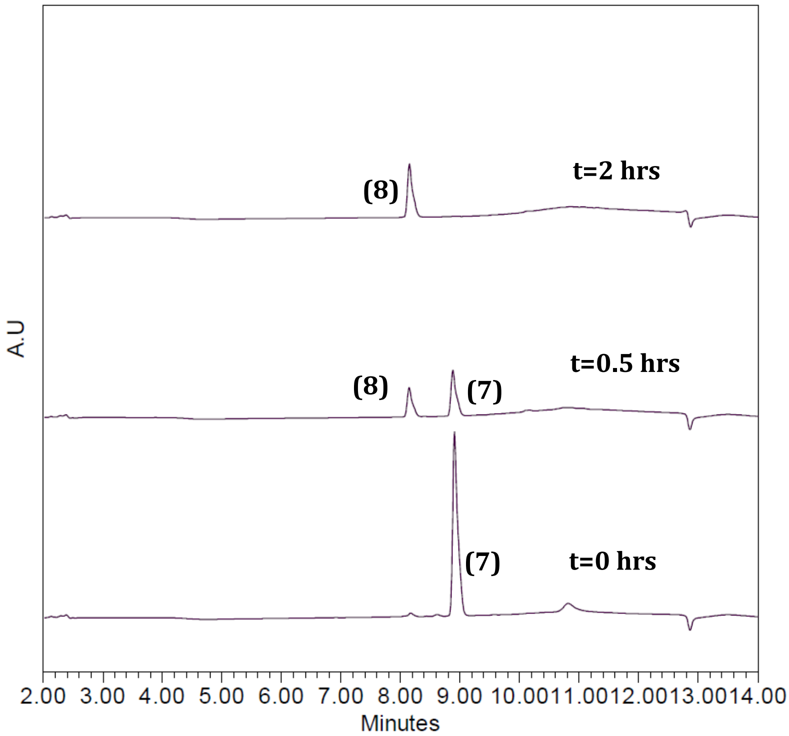

Figure S29- *Representative HPLC chromatogram overlay (taken at 252 nm), showing the transformation of HexylBPE substrate (7) to its' de-propargylated product (8) in the presence of Pd(OAc)$_2$ loaded micelles made of mPEG$_{5k}$-D-(C14)$_4$ amphiphiles.*

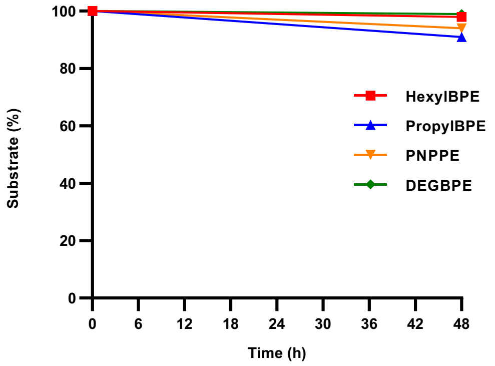

Figure S30 *– Substrate stability in the presence of C14 micellar solution, without the addition of Pd(OAc)2 salt.*

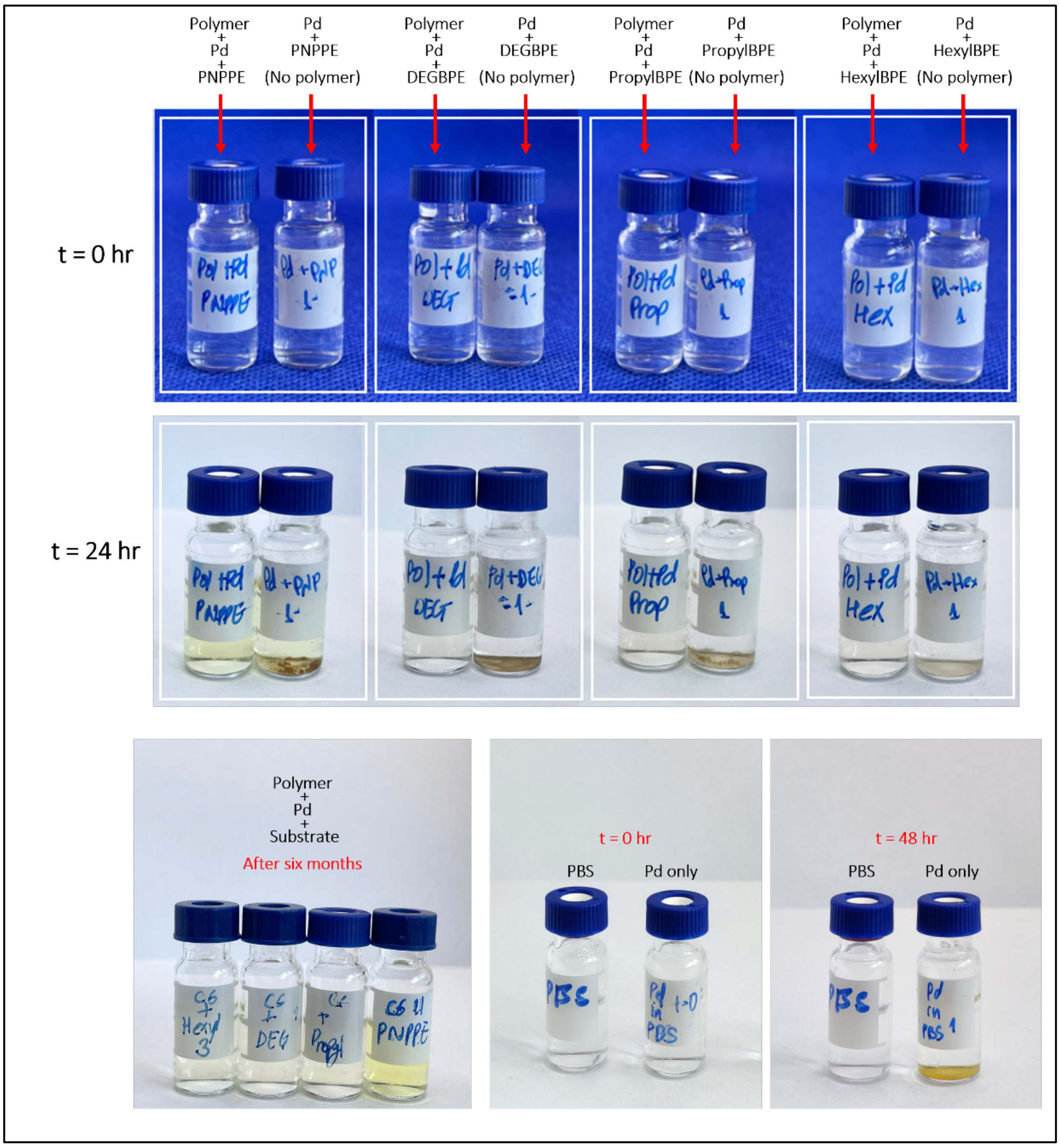

Figure S31 *– Stability of Pd(OAc)2 salt in aqueous solution in the presence or absence of C6 micelles.*

ICP-MS

Sample preparation: The amphiphiles and metal solutions were prepared as described before. $Pd(OAc)_2$ solution in the absence of micelles was prepared by directly dissolving the metal salt in PBS, following by vortex and sonication to afford clear solution.

Dialysis Experiments: 200 μL of sample was loaded into a Mini GeBAflex Tube having molecular weight cutoff of 8 kDa. These tubes were placed in Corning® 15 mL centrifuge tubes containing 10 mL PBS which was kept in an incubator at 37 °C for 24 hours and shaken at 90 rpm. After 24 hours, samples were taken out from the dialysis tubes and from The dialysis experiments were repeated three times.

ICP-MS measurements: 100 μL of inner sample solution or 1 mL of outer sample solutions were mixed with 900 μL of HNO_3 and 200 μL of H_2O_2 and were heated to 95 °C for 15 min. The solutions were further diluted with 8.8 mL of water. Measurements were done on 7800 ICP-MS equipped with SPS4 Autosampler (Agilent). The instrument was operated in Helium mode with "General Conditions" plasma. Signal of 105 Pd isotope was measured with 6 replicates per measurement.

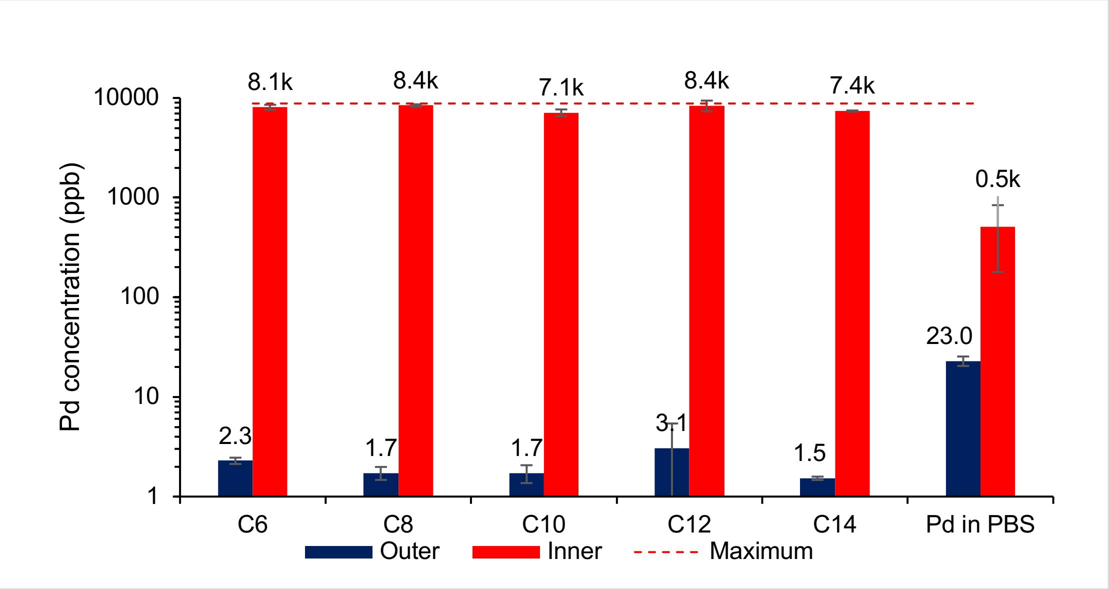

Figure S32 *– ICP-MS analysis of Pd in the presence of CX amphiphiles or PBS only, after dialysis for 24 hr. Both inner solutions from dialysis tubes (red) and outer solutions (blue) were characterized. The expected concentration of Pd based on the added amount of Pd= 8,750 ppb (marked as dashed line).*

* The total calculated concentration for the Pd solutions in the absence of micelles is lower than expected due precipitation of the metal or interactions with the dialysis membrane.

General protocol for the Na_2PdCl_4 palladium mediated depropargylation reaction experiments setup

Na_2PdCl_4 was dissolved in PBS to yield final concentration of 83 μM. C14 amphiphiles were directly added to this solution, allowing the formation of the C14 micellar nanoreactor, with final concentrations of 83 μM and 42 μM for the metal salt and the amphiphile, respectively.

To initiate the reaction, the substrates were added to the aqueous nanoreactor solution, as described above for the $Pd(OAc)_2$ experimental protocol.

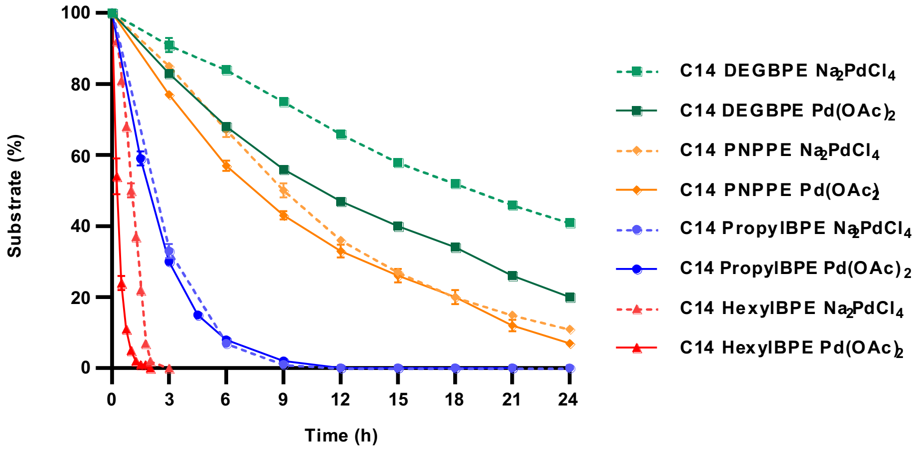

Figure S33- *Normalized substrate consumption over time, in the presence of C14 nanoreactors using Na_2PdCl_4 salt (dashed lines) and $Pd(OAc)_2$ salt (full lines). [C14 Amphiphile] = 42 μM; [Metal] = 83 μM; [Substrate] = 100μM for HexylBPE, PropylBPE, DEGBPE and 160μM for PNPPE.*

General protocol for the palladium mediated depropargylation reaction in Acetone:PBS mixture experiments setup

Stock solutions of $Pd(OAc)_2$ was made in acetone at a concentration of 166 μM, then diluted with PBS in a 1:1 v/v ratio to yield final concentration of 83 μM for the metal salt.

Stock solutions of the substrates were made separately in DMSO as mentioned and were added to the PBS:Acetone metal solution following the same protocol as for the micellar nanoreactors solutions.

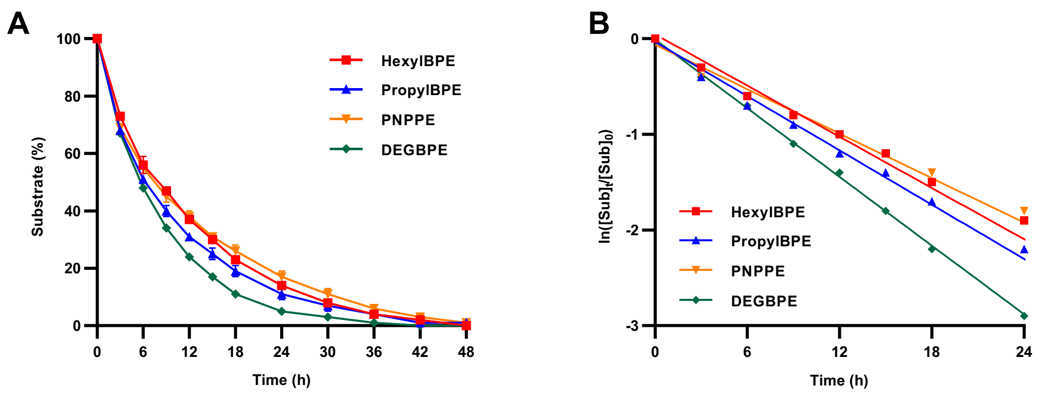

Figure S34- *O-Propargyl cleavage profile of Hexyl, Propyl, PNPPE and DEG substrates in Acetone+PBS (1:1 v/v) set-up* ***A)*** *Normalized substrate consumption over time.* ***B)*** *Natural log of the normalized experimental kinetic data*

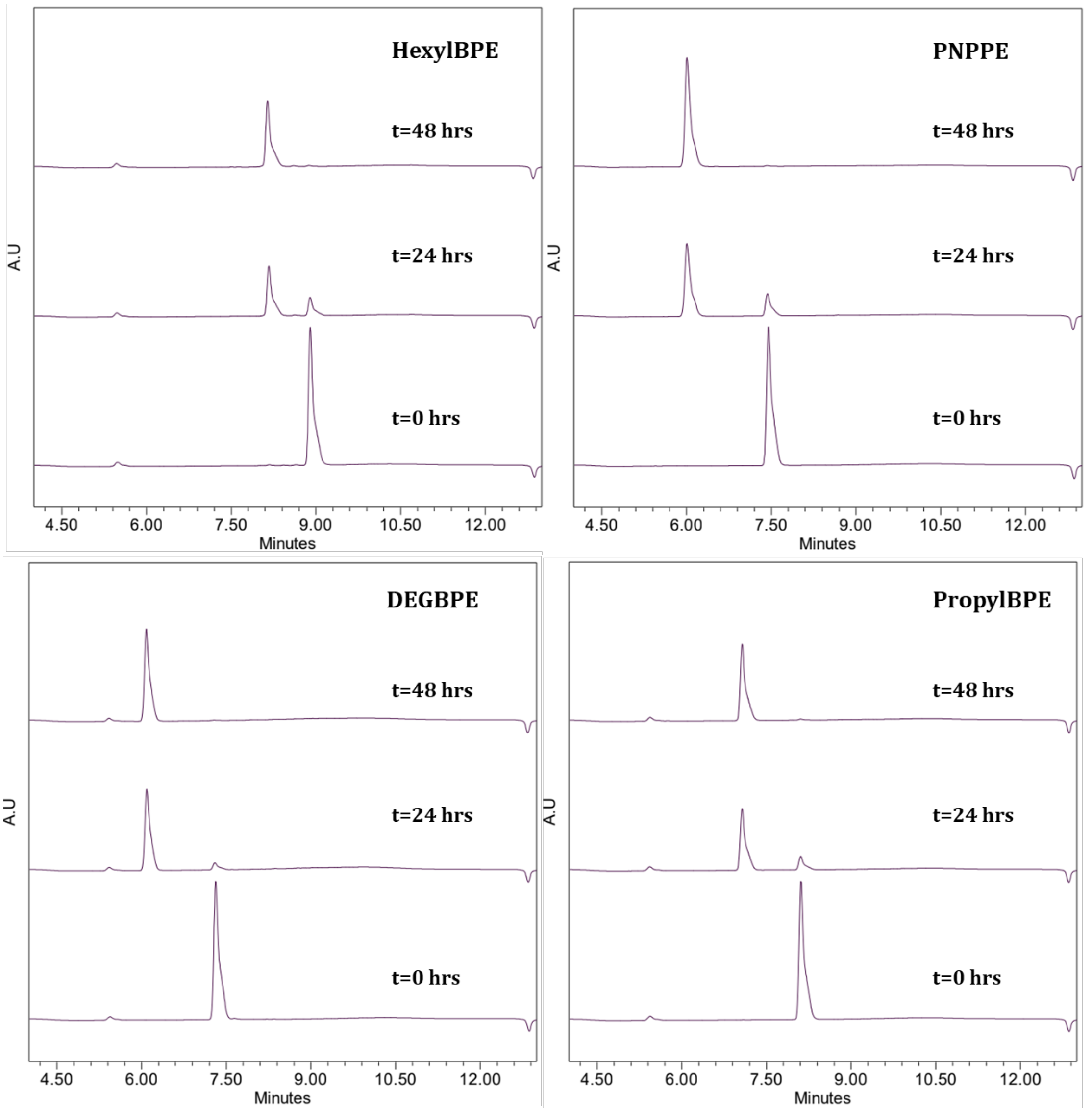

Figure S35- *Representative HPLC chromatogram overlay, showing the transformation of propargylated substrates to their de-propargylated product in the presence of* $Pd(OAc)_2$ *in an Acetone: PBS (1:1, v/v) mixture.*

References

(1) Harnoy, A. J.; Buzhor, M.; Tirosh, E.; Shaharabani, R.; Beck, R.; Amir, R. J. Modular Synthetic Approach for Adjusting the Disassembly Rates of Enzyme-Responsive Polymeric Micelles. *Biomacromolecules* **2017**, *18* (4), 1218–1228. https://doi.org/10.1021/acs.biomac.6b01906.
